# High-frequency repetitive transcranial magnetic stimulation (rTMS) protects against ischemic stroke by inhibiting M1 microglia polarization through let-7b-5p/HMGA2/NF-κB signaling pathway

**DOI:** 10.1186/s12868-022-00735-7

**Published:** 2022-08-04

**Authors:** Ye Hong, Jinfeng Lyu, Lin Zhu, Xixi Wang, Mengna Peng, Xiangliang Chen, Qiwen Deng, Jie Gao, Zhenhua Yuan, Di Wang, Gelin Xu, Mengyi Xu

**Affiliations:** 1 Department of Neurology, Nanjing First Hospital, Nanjing Medical University, 68# Changle Road, Nanjing, 210029 Jiangsu China; 2 Department of Neurology, Jinling Hospital, Medical School of Nanjing University, Nanjing, 210002 Jiangsu China

**Keywords:** Ischemic stroke, Microglia polarization, rTMS, Let-7b-5p, HMGA2, NF-κB

## Abstract

**Background:**

Microglia assume opposite phenotypes in response to ischemic brain injury, exerting neurotoxic and neuroprotective effects under different ischemic stages. Modulating M1/M2 polarization is a potential therapy for treating ischemic stroke. Repetitive transcranial magnetic stimulation (rTMS) held the capacity to regulate neuroinflammation and astrocytic polarization, but little is known about rTMS effects on microglia. Therefore, the present study aimed to examine the rTMS influence on microglia polarization and the underlying possible molecular mechanisms in ischemic stroke models.

**Methods:**

Previously reported 10 Hz rTMS protocol that regulated astrocytic polarization was used to stimulate transient middle cerebral artery occlusion (MCAO) rats and oxygen and glucose deprivation/reoxygenation (OGD/R) injured BV2 cells. Specific expression levels of M1 marker iNOS and M2 marker CD206 were measured by western blotting and immunofluorescence. MicroRNA expression changes detected by high-throughput second-generation sequencing were validated by RT-PCR and fluorescence in situ hybridization (FISH) analysis. Dual-luciferase report assay and miRNA knock-down were applied to verify the possible mechanisms regulated by rTMS. Microglia culture medium (MCM) from different groups were collected to measure the TNF-α and IL-10 concentrations, and detect the influence on neuronal survival. Finally, TTC staining and modified Neurological Severity Score (mNSS) were used to determine the effects of MCM on ischemic stroke volume and neurological functions.

**Results:**

The 10 Hz rTMS inhibited ischemia/reperfusion induced M1 microglia and significantly increased let-7b-5p level in microglia. HMGA2 was predicted and proved to be the target protein of let-7b-5p. HMGA2 and its downstream NF-κB signaling pathway were inhibited by rTMS. Microglia culture medium (MCM) collected from rTMS treated microglia contained lower TNF-α concentration but higher IL-10 concentration than no rTMS treated MCM, reducing ischemic volumes and neurological deficits of MCAO mice. However, knockdown of let-7b-5p by antagomir reversed rTMS effects on microglia phenotype and associated HMGA/NF-κB activation and neurological recovery.

**Conclusion:**

High-frequency rTMS could alleviate ischemic stroke injury through inhibiting M1 microglia polarization via regulating let-7b-5p/HMGA2/NF-κB signaling pathway in MCAO models.

**Supplementary Information:**

The online version contains supplementary material available at 10.1186/s12868-022-00735-7.

## Introduction

Microglia are the resident macrophages and the main cell type to participated in acute and chronic neuroinflammatory reactions following brain injuries [1, 2]. Besides, growing evidences have demonstrated that microglia was highly plastic cells with diverse phenotypes and biphasic roles in response to different stimuli [3]. Classically activated M1 microglia release proinflammatory mediators to exert a deleterious role; alternatively activated M2 phenotype release anti-inflammatory and neurotropic medicators to attenuate neuronal injury [4]. Both types of microglia were reported in cerebral ischemic animals, undergoing an early ‘beneficial’ M2 phenotype and then shifted to a ‘detrimental’ M1 phenotype in the peri-infarct regions [3, 5]. Preventing M1 formation, promoting M1 reversion, or blocking the M1 neurotoxin holds great potential for alleviating neuroinflammatory injury after cerebral ischemic stroke.

Repetitive transcranial magnetic stimulation (rTMS) is a noninvasive brain-stimulating technique with attractive treating effects on ischemic stroke patients [6, 7]. The classic mechanism of rTMS therapy is to regulate neural activity, but some studies have shown that rTMS could reduce neuroinflammation, and glial cells could be the likely effectors of rTMS stimulation [8–11]. Our previous study demonstrated that the 10-Hz rTMS inhibited neurotoxic polarization of astrocytes after focal cerebral ischemia [12]. However, it’s not clear whether and how the 10-Hz rTMS affects microglia.

In the current study, we explored the 10-Hz rTMS effects on microglia polarization in both in vitro and in vivo cerebral ischemic models. iNOS was chosen as M1 microglia marker and CD206 was used to label M2 microglia. As microglia polarization is closely related to different miRNA expression levels and rTMS has profound effects to regulate miRNA levels [13–15], the miRNA and associated signaling pathway changes induced by rTMS in microglia were also detected. Our results demonstrated that the 10-Hz rTMS could inhibit M1 polarization of microglia to promote functional recovery after ischemic stroke, and the underlying mechanism was associated with let-7b-5p related signaling pathway. As far as we know, it is the first study to investigate the rTMS impacts on microglia polarization and its possible mechanisms in cerebral ischemic stroke models.

## Materials and methods

### Focal cerebral ischemia

Male Sprague–Dawley rats (250–270 g) and C57BL/6 J mice (20–25 g) were reared under a 12-h light/dark cycle at approximately 25 °C and 65% humidity, and were provided with free access to food and water. Transient focal cerebral ischemia was induced by middle cerebral artery occlusion (MCAO) surgery following previous methods [12, 16]. A silicon-coated monofilament (diameter 0.18 mm for mice, 0.36 mm for rats) was used to occlude the right middle cerebral artery for 90 min and subsequently withdrawn for reperfusion. A heating pad was used to maintain the body temperature at 37.0 ± 0.5 °C during surgery. The sham-operated animals underwent the same procedures except that the middle cerebral artery was not occluded. A total of 42 male rats (n = 8 for sham operation; n = 34 for MCAO) and 46 male mice (n = 8 for sham operation; n = 38 for MCAO) were used in this study. All animal experiments were carried out in compliance with the ARRIVE guidelines (Animal Research: Reporting of In Vivo Experiments) and approved by the ethical standards of Nanjing First Hospital (Permit Number: DWSY-2104285). Maximum efforts have been made to minimize the number of animals used and their suffering.

### Primary neuron culture

Primary cortical neurons were isolated from the embryonic *C57BL/6 J* mice (E16–18) according to previous reports [12]. Briefly, cortical tissues were digested with 0.125% trypsin (Gibco, MD, USA) for 15 min. Then the cortical tissues were pipetted blow and mixed 100 times and filtered with a 100-μm cell strainer (Biologix, Shandong, China) to remove impurities. Subsequently, the cells were centrifuged and suspended in DMEM medium (Hyclone, UT, USA) with 10% FBS (Gibco, USA) and then seeded into flasks. Two hours later, the DMEM medium was replaced with neurobasal medium supplemented with 2% B27 and 1% glutamax (Thermo Fisher Scientific, MA, USA).

### BV-2 microglia culture and OGD/R

BV-2 microglial cell line, purchased from China Infrastructure of Cell Line Resources (Beijing, China), was grown in DMEM F12 medium (Gibco, USA) supplemented with 10% FBS (Invitrogen, USA), and 1% penicillin–streptomycin (Invitrogen, USA). oxygen and glucose deprivation/reoxygenation (OGD/R) was conducted according to a previously established protocol [17]. Normal cell culture medium was removed and changed by glucose-free DMEM medium (Gibco, USA). Then, cells were incubated in an oxygen-free chamber equipped with AnaeroPack-Anaero (MGC, Japan) at 37 °C for 2 h. Finally, the cells were returned to normal incubator and incubated with the initial culture medium for reoxygenation.

### rTMS

The 10 Hz repetitive transcranial magnetic stimulation for BV2 microglial cells and MCAO rats were conducted according to a previous study [12]. A customized magnetic stimulator (MagPro X 100 with Magoption, Tonica, DK) with a C-100 circular coil (20 mm in inner diameter with 1.9 T peak magnetic stimulator output) was used. For BV2 cells, the magnetic coil was placed and positioned 1 cm away from the cell culture dish to give 10 Hz for two consecutive days. For MCAO rats, rTMS started at 24 h after the reperfusion and lasted for 7 days. The magnetic coil was located above the ipsilateral primary motor cortex (right M1 region). The rTMS stimulation paradigm consisted of stimulation for 3 s followed by 50 s of rest, which was repeated twenty times (600 pulses per day) at a frequency of 10 Hz.

### Cerebral infarct volume

The 2, 3, 5-triphenyltetrazoliumchloride (TTC, Sigma, USA) staining was applied to detect infarct volume. After the mice were sacrificed, their brains were cut into 1-mm sections immediately. Then, the brain slices were incubated with 2% TTC solution at 37 °C for 15 min and fixed by 4% paraformaldehyde (PFA) at 4 °C overnight. The relative infarct volume was calculated as previously reported [12].

### Neurological deficit evaluation

Neurological deficits of the experimental animals were assessed with the modified neurologic severity score (mNSS) as described [18]. The mNSS scoring system consists of four tests: motor, sensory, balance, reflex tests and abnormal movements, such as seizure. The total scores ranged from 0 to 18, which 0 represents no deficit and 18 represents maximal deficit.

### Immunofluorescence

Samples were fixed with 4% paraformaldehyde for 30 min, and permeabilized with 0.1% Triton X-100 for 10 min, then blocked with 5% BSA in PBS for 60 min at 37 °C and incubated with the indicated primary antibodies at 4 °C overnight. Primary antibodies include: anti-Iba-1 (1:200; # ab178847, Abcam, UK), anti-iNOS (1:100; # ab210823, Abcam, UK), anti-CD206 (1:100; #sc-376232, Santa Cruz, USA). After four washes with PBS, the samples were incubated with corresponding fluorescence-conjugated secondary antibodies (1:500, Jackson Immuno Research, USA) for 2 h, followed by DAPI (Sigma-Aldrich, USA) staining for 10 min. Finally, samples were observed under a FluoView FV10i confocal laser scanning microscope (Olympus, Japan) or an Olympus BX51 microscope. The fluorescently-stained cells were analyzed by Image J software (NIH, USA).

### Fluorescence in situ hybridization (FISH)

FISH was performed in tissue sections using fluorescence in situ hybridization (FISH) kit (Servicebio, Wuhan, China) and the let-7b-5p detection probe (Servicebio, Wuhan, China) by following the manufacturer’s protocol.

### Western blot analysis

Western blot analysis was conducted according to previous report [12]. Proteins were extracted from cortical tissues or cultured cells with RIPA lysis buffer (Cell Signaling Technology, MA, USA). The protein concentrations were detected by the BCA protein assay kit (Generay Biotechnology, Shanghai, China). Then, equal amount of protein was applied for SDS-PAGE electrophoresis and transferred to polyvinylidene difluoride (PVDF) membranes (Millipore, MA, USA). After blocking with 10% skim milk for 1 h, the PVDF membranes were cut according to the molecular weight of the proteins as indicated in the instruction manuals, then incubated with primary indicated antibodies: CD206 (1:500; #sc-376232, Santa Cruz, USA), iNOS (1:1000; # ab210823, Abcam, UK), HMGA2(1:1000; ab97276, Abcam, UK), p-IκBα(1:1000, #2859, Cell Signaling Technology, USA), IκBα (1:1000, #9242, Cell Signaling Technology), p-P65(1:1000, #3036, Cell Signaling Technology), P65(1:1000, #4764, Cell Signaling Technology), and β-actin (1:5000, Cell Signaling Technology) overnight at 4 °C. After four washes with PBST, HRP-conjugated secondary antibodies were used for further incubation of the membranes for 120 min at room temperature. Finally, the protein signals were developed by the ECL solution (Millipore, MA, USA). Quantitative analysis of protein blots was analyzed by Image J software. The original unedited blots were presented in Additional file 2.

### miRNA sequencing and analysis

Peri-infarct cortex tissue derived from the MCAO and MCAO + rTMS rats (n = 3) were used for miRNA sequencing. The miRNA sequencing was completed by Illumina HiSeqTM 2500 platform and data analysis was performed by Ribo Bio (Co., Ltd., Guangdong, China). Comparisons between groups were performed with two-tailed Student’s t-tests, wherein *P* < 0.05 and fold changes > 2 were considered significant.

### Quantitative real-time polymerase chain reaction (qRT-PCR)

Total RNA was extracted from peri-infarct cortex tissues and BV2 cells using TRIzol regent (Sigma-Aldrich, USA) followed by cDNA synthesized by RevertAid First Strand cDNA Synthesis Kit (Thermo Fisher Scientific, USA). SYBR Green based quantitative PCR was conducted on a Stratagene Mx3000P real-time PCR system (Agilent Technologies). U6 was used as endogenous controls for miRNA, results were calculated using 2^−ΔΔCt^ (Ct, threshold cycle). Primers for qRT-PCR were listed in Additional file 3: Table S1.

### miRNA transfection and dual-luciferase reporter assay

Let-7b-5p agomir, antagomir, the entire and mutated 3’ UTR of HMGA2 were generated by Ribo Bio (China). For miRNAs transfection, 293 T or BV2 cells were incubated with 50 nM let-7b-5p agomir, antagomir or non-targeting control sequence using Lipofectamine 3000 Reagent (Thermo Fisher Scientific, USA). Dual-luciferase report assay was conducted according to previous report [17]. Briefly, 293 T cells were plated into 96-well plates and cultured to 90–95% confluence. Cells in each well were then co-transfected with 1 µg of the indicated 3′ UTR luciferase reporter vectors and 100 nM of let-7b-5p agomir or control agomir (Ribo Bio, China). Finally, cells were harvested after 48 h to measure the Renilla and firely luciferase activities by the Dual-Luciferase Reporter Assay System (Promega, MI, USA).

### Microglia-conditioned media collection

BV2 microglia-conditioned media (MCM) were obtained as previously reported [12]. After OGD/R and receiving rTMS stimulation, the BV2 culture media were collected at 48 h post-OGD and centrifuged. The MCM were applied to ELISA experiment or used to co-culture neurons.

Concentrated MCM was used for MCM therapy. The concentrated MCM were obtained by centrifugation of MCM in the 10 KDa-membrane centrifuge tubes (Millipore, UFC901024) at 4000*g* at 21 °C for 30 min.

### MCM Therapy

Under anesthesia, each mouse received posterior orbital intravenous injection of 100 μL concentrated MCM at the time of MCAO reperfusion, and at three days post reperfusion.

### Enzyme-linked immunosorbent assay (ELISA)

The concentrations of TNF-α and IL-10 in all MCM samples were measured by the specific ELISA kit (Neobioscience, China). These measurements were based on the instructions of manufacturer.

### Cell death detection

Propidium iodide (PI)/Hoechst 33342 assay kit (Thermo Fisher Scientific, USA) was used to detect cell death following the manufacturer’s instructions. Firstly, PI/Hoechst 33342 solution was added to the neurons which were cultured with different MCM for 48 h. After incubating for 15 min, the neurons were washed with PBS for five times and observed under an Olympus BX51 microscope. The dead cells were stained with propidium iodide (red), while live cells stained with Hoechst 33342 (blue). The fluorescently-stained cells were calculated by Image J software (NIH, USA).

### Statistical analysis

*SPSS 22.0* software (IBM, Armonk, NY, USA) was used for data analysis. Differences between groups were compared using two-tailed Student’s *t* tests and one-way ANOVA followed by Tukey’s post hoc test. All data are expressed as mean ± SD. Statistical significance was determined as *P* < 0.05.

## Results

### rTMS inhibited M1 microglial polarization induced by ischemia–reperfusion

**Fig. 1:**
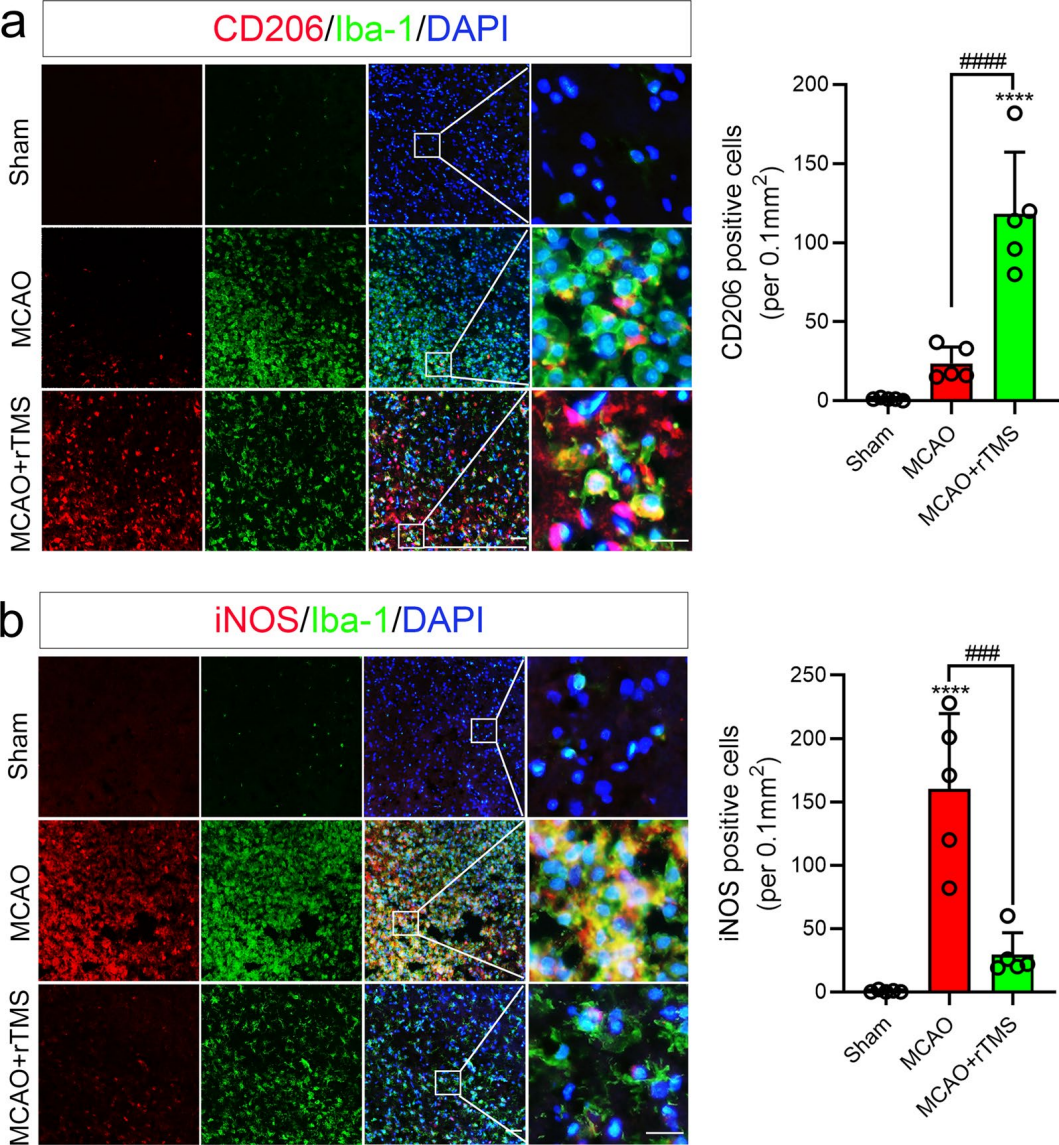
The 10 Hz rTMS reversed cerebral ischemia–reperfusion induced microglial polarization. **a** Co-staining and quantification of CD206^+^/Iba-1^+^ cells; **b** co-staining and quantification of iNOS^+^/Iba-1^+^ cells in peri-infarct zone at 7 days after MCAO reperfusion. Magnified views of merged staining are marked with solid line boxes. n = 5. Data are expressed as mean ± SD; ****P < 0.0001 versus sham group; ^####^*P* < 0.0001 versus MCAO group. Scale bar: 10 μm

Microglia has been proven to respond dynamically to ischemic injury in mice MCAO model, undergoing an M2-to-M1 phenotype shift [5]. Similar dynamic phenotype transformation was also observed in microglia in the rat MCAO model (Additional file 1: Fig. S1). M2 marker CD206 was significantly increased at 1 day after MCAO and declined from day 5 onward (Additional file 1: Fig. S1a), while M1 marker iNOS was significantly increased from day 3 to day 14 after ischemic reperfusion (Additional file 1: Fig. S1b). Previously reported 10-Hz rTMS protocol was applied to the ipsilateral hemisphere of MCAO rats for seven days, then the polarization phenotype of microglia in the peri-infarct region was detected by immunofluorescent staining. As shown in Fig. 1a, Iba-1 signaling was increased in both the ischemic core and peri-infarct region in MCAO rats with or without rTMS treatment at 7 days post ischemia–reperfusion, which indicated activation of microglia. However, more activated microglia were co-stained with M2 marker CD206 in MCAO + rTMS group (118.4 ± 38.87 versus 23.60 ± 10.53 per 0.1 mm^2^, *P* < 0.001), while less co-stained with M1 marker iNOS (29.40 ± 17.31 versus 160.4 ± 59.39 per 0.1 mm^2^, *P* < 0.001) than in MCAO group (Fig. 1). Above results indicated that the 10 Hz rTMS hold the ability to reverse M1 polarization of microglia induced in cerebral ischemic stroke model.

### rTMS counteracted OGD/R induced microglial polarization and associated neurotoxic effects

**Fig. 2:**
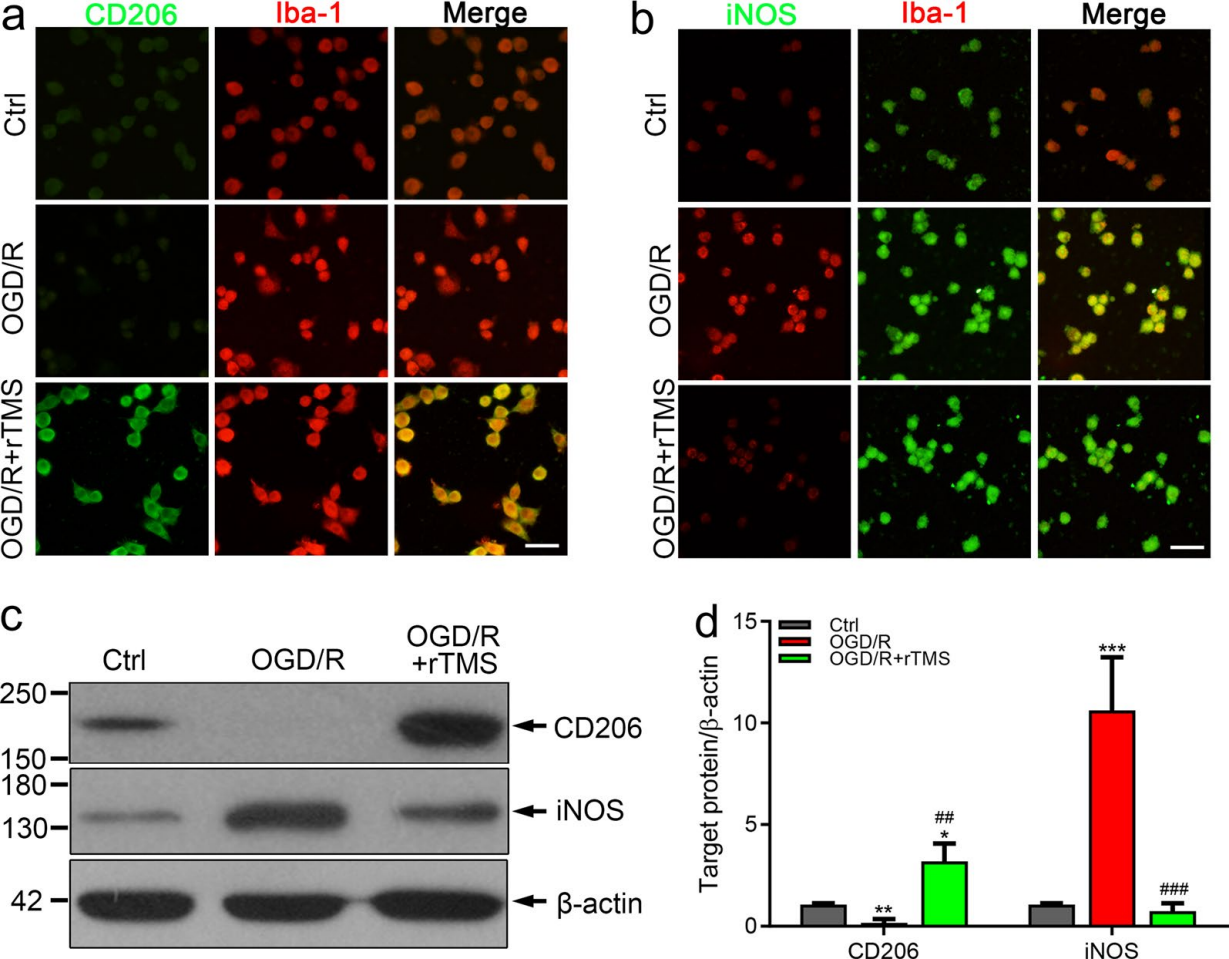
The 10 Hz rTMS inhibited OGD/R injury induced M1 polarization of microglia. Immunostaining for CD206 (**a**) and iNOS (**b**) in BV2 cells with indicated treatment. **c**, **d** Western blots and quantification of CD206 and iNOS in each group. n = 3. Data are expressed as mean ± SD; *P < 0.05, **P < 0.01, ***P < 0.001 versus Ctrl group; ^##^*P* < 0.01, ^###^*P* < 0.001 versus OGD/R group. Scale bar: 10 μm

**Fig. 3:**
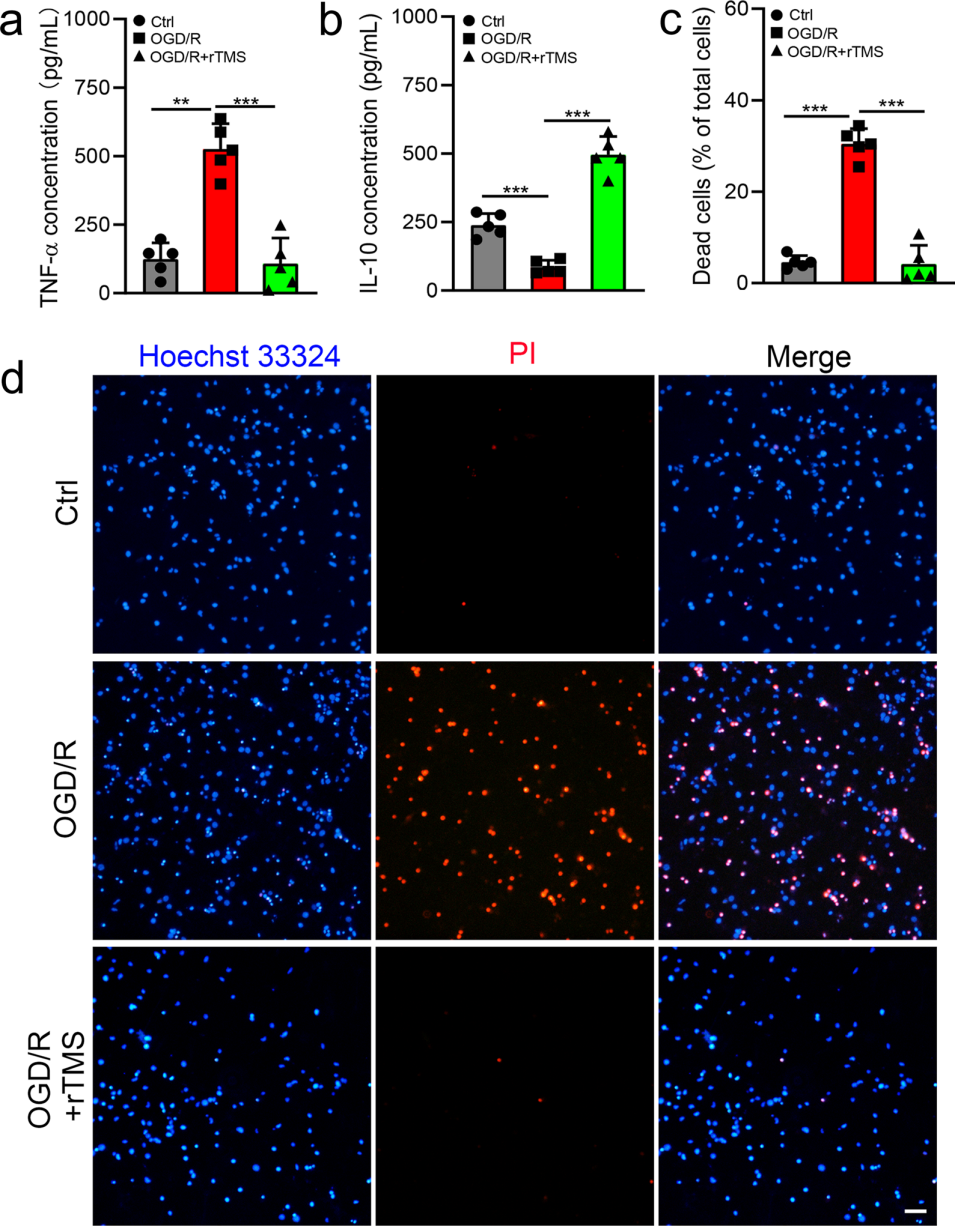
The 10 Hz rTMS alleviated neurotoxicity of microglia after OGD/R injury. **a** TNF-α concentration and **b** IL-10 concentration in Ctrl MCM, OGD/R MCM and OGD/R + rTMS MCM. **c**, **d** Neurons were co-cultured with Ctrl MCM, OGD/R MCM and OGD/R + rTMS MCM for 48 h, PI/Hoechst staining was applied to detect vitality of neurons. Dead neurons were positively stained with red PI (**d**) and dead neurons were quantified by Image J (**c**). n = 5. Data are expressed as mean ± SD; **P < 0.01, ***P < 0.001. MCM, microglia culture media. Scale bar: 10 μm

In MCAO models, many kinds of cells have been reported to be regulated by rTMS, it was not clear whether the polarization of microglia is directly regulated by rTMS or the secondary effects induced by other cells. We applied rTMS to a cultured microglial cell line, named BV2 cells to explore the potential of rTMS to directly regulate microglia. After OGD/R injury, the fluorescent signal of M2 marker CD206 was weakened (Fig. 2a), while signal of M1 marker iNOS was significantly increased (Fig. 2b), indicating M1 polarization of microglia. After receiving rTMS stimulation, CD206 signals was enhanced and iNOS signals was reduced (Fig. 2a and b). The western blotting results were consistent with the immunofluorescent staining results (Fig. 2c and d). In OGD/R BV2 cells, CD206 was 5.29-fold lower (0.17 ± 0.18 versus 1.07 ± 0.07, *P* = 0.001) and iNOS was 10.21-fold higher (10.62 ± 2.61 versus 1.04 ± 0.10, *P* < 0.001) than those in control cells. In rTMS treated OGD/R BV2 cells, CD206 was increased to 2.99-fold (3.20.880 ± 0.39 versus 1.07 ± 0.07,) and iNOS not significantly altered when compared to the control cells (0.74 ± 0.39 versus 1.04 ± 0.10, *P* = 0.963). Indicating that microglia polarized to M1 phenotype after OGD/R injury, while rTMS treatment promoted M2 transition of microglia. M1 microglia could secret proinflammatory mediators, while M2 microglia secret anti-inflammatory and neurotrophic mediators. Thus, we collected microglia culture media (MCM) from different groups, and detect the concentrations of pro-inflammatory mediator TNF-α and anti-inflammatory IL-10. As shown in Fig. 3a, the concentration of TNF-α in OGD/R MCM was 4.24-fold higher than Ctrl MCM (526.2 ± 91.93 versus 124.1 ± 59.26 ng/mL, *P* < 0.001), while rTMS treatment reduced TNF-α to 107.5 ± 93.90 ng/mL (*P* = 0.95 versus Ctrl MCM, *P* < 0.001 versus OGD/R MCM). Besides, the concentration of IL-10 in OGD/R MCM was 35.9% of Ctrl MCM (85.75 ± 24.44 versus 238.8 ± 42.15 ng/mL, *P* = 0.0007), while rTMS increased IL-10 to 496.1 ± 66.67 ng/mL (*P* < 0.001 versus Ctrl MCM, *P* < 0.001 versus OGD/R MCM) (Fig. 3b). We then mixed MCM with neuronal culture media to incubate neurons and explored the indirectly effects of rTMS to neurons through regulating microglia. As shown in Fig. 3c and d, the most neuron death was detected in OGD/R MCM cultured group (4.59 ± 1.44%, 30.47 ± 3.33%, 4.19 ± 4.09% neuronal death, respectively for Ctrl, OGD/R, OGD/R + rTMS groups, *P* < 0.001), and no difference was found between control group and OGD/R + rTMS group (*P* = 0.97). Above data indicated that rTMS could inhibit OGD/R induced M1 polarization of microglia and alleviate microglia associated neurotoxic effects.

### rTMS altered microglial let-7b-5p expression flowing ischemia–reperfusion

**Fig. 4:**
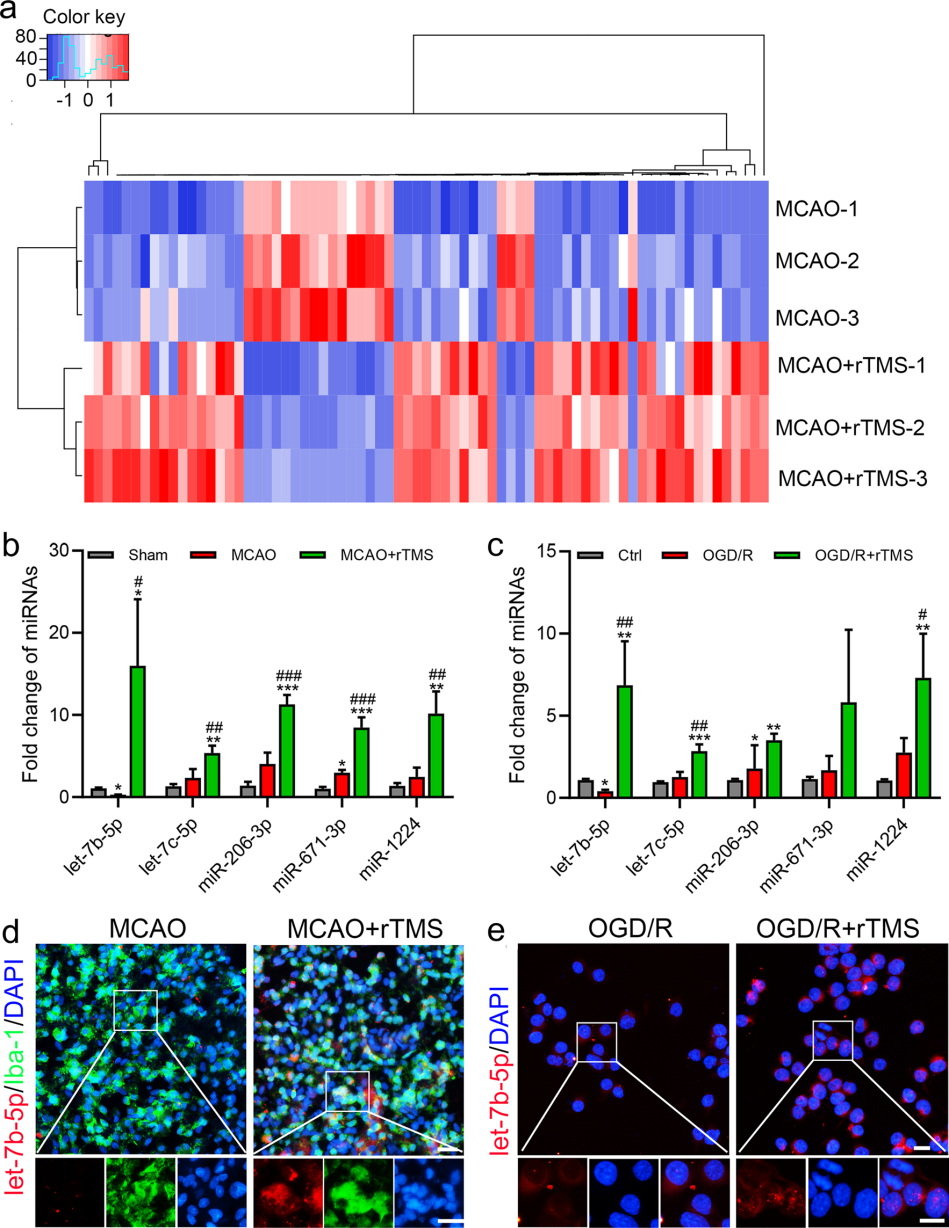
The 10 Hz rTMS increased microglial expression of let-7b-5p after ischemia–reperfusion and OGD/R injury. **a** Heatmap of the microRNAs with at least two-fold change and CT value > 200 in expression in cerebral peri-infarct tissues. **b** Real-time PCR for validation of miRNAs significantly increased by the 10 Hz rTMS treatment in the cerebral peri-infarct tissues of MCAO rats. **c** Real-time PCR for validation of the five miRNAs in OGD/R BV2 cells. **d** Combined FISH and IF for let-7b-5p (red) and Iba-1 (green) in the peri-infarct region of MCAO rats. **e** FISH for the staining of let-7b-5p in OGD/R BV2 cells. n = 3. Data are expressed as mean ± SD; *P < 0.05, **P < 0.01, ***P < 0.001 versus Sham or Ctrl group; ^#^*P* < 0.05, ^##^*P* < 0.01, ^###^*P* < 0.001 versus MCAO or OGD/R group. Scale bar: 10 μm

To examine possible changes of miRNAs in response to the rTMS treatment after MCAO, high-throughput miRNA sequencing was performed. Seventy-three miRNAs were found with CT value > 200, fold change ≥ 2, and *P* < 0.05 between MCAO group and MCAO + rTMS group (Fig. 4a and Additional file 4: Table S2). Five of the top six miRNAs (let-7b-5p, let-7c-5p, miR-206-3p, miR-671-3p, and miR-1224) increased by rTMS treatment were conserved in rat and mouse, except for the miR-485-3p (mature sequences of miRNAs were listed in Additional file 5: Table S3). Real-time polymerase chain reaction was applied to validated changes of these five miRNAs in both brain tissues and BV2 cells. In the peri-infarct region, rTMS significantly increased these miRNA expressions when compared to Sham group and MCAO group, with let-7b-5p increased the most (Fig. 4b). The same expression tendencies of let-7b-5p, let-7c-5p, and miR-1224 were also detected in BV2 cells, while rTMS increase of miR-206-3p and miR-671-3p were not significant when compared to OGD/R injured BV2 cells (Fig. 4c). Besides, it was interesting that only let-7b-5p was decreased when compared MCAO group with sham group (Fig. 4b), and compared OGD/R group with Ctrl group (Fig. 4c), while the other four miRNAs were increased or remained unchanged. So, we chose to explore the influence of rTMS on let-7b-5p in microglia. miRNA fluorescent in situ hybridization (FISH) was applied and a substantial increase of let-7b-5p was found in microglia in the peri-infarct cortex (Fig. 4d) and OGD/R BV2 cells after rTMS treatment (Fig. 4e). In conclusion, rTMS could increase let-7b-5p expression in microglia after I/R and OGD/R injury.

### rTMS effects on let-7b-5p/HMGA2/NF-κB pathway

**Fig. 5:**
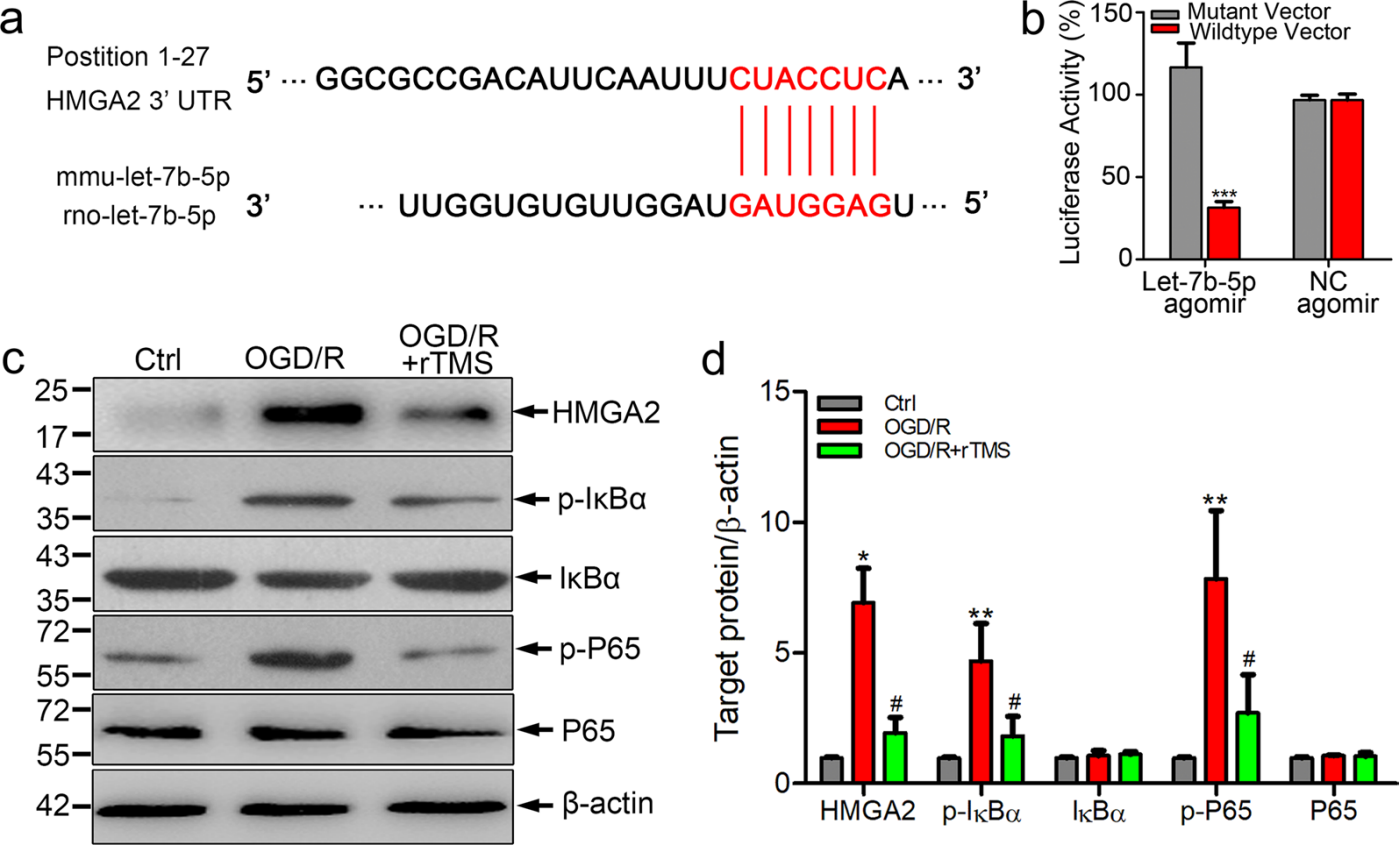
Let-7b-5p regulated HMGA2 expression in BV2 cells. **a** The predicted binding sites of let-7b-5p to 3′-UTR of *HMGA2* gene. **b** Dual luciferase reporter assay of the relationship between let-7b-5p agomir and wild type 3′ UTR of *HMGA2*. **c**, **d** Western blots and quantification of HMGA2, p-IκBα, IκBα, p-P65 and P65 in BV2 cells with indicated treatment. n = 3. Data are expressed as mean ± SD; *P < 0.05, **P < 0.01 versus Ctrl group; ^#^*P* < 0.05 versus OGD/R group

Since the biological functions of miRNAs rely on downstream target genes, bioinformatic algorithm (TargetScan) was applied to analyze the potential targets of let-7b-5p. As shown in Fig. 5a, HMGA2 was a predicted downstream target gene of let-7b-5p. Dual-luciferase reporter assays were conducted to ascertain whether let-7b-5p could specifically recognize the 3′ UTR of *HMGA2*. Transfection of let-7b-5p agomir substantially inhibited the activity of *HMGA2* 3′ UTR compared to negative control transfected cells (*P* < 0.001, Fig. 5b). On the contrary, when the let-7b-5p binding site in the 3′ UTR of *HMGA2* was mutated, luciferase activities were significantly depressed. Above data demonstrated that HMGA2 was a potential functional target of let-7b-5p in microglia.

HMGA2 could activate NF-κB-medicated inflammatory response through regulating PI3K/Akt signaling and NF-κB-associated signaling implicated in the M1 microglial polarization process [19, 20]. To explore whether rTMS had effects on HMGA2/NF-κB pathway, western blotting analysis was carried out to detect HMGA2 expression and activation of NF-κB in OGD/R BV2 cells with and without rTMS treatment. As expected, OGD/R injury remarkably induced the expressions of HMGA2, p-IκBα and p-P65 in BV2 cells, while rTMS inhibited HMGA2 expression and caused significant decreases of p-P65 and p-IκBα (Fig. 5c and d). In conclusion, HMAG2 was a downstream target of let-7b-5p, rTMS could reverse OGD/R induced change of let-7b-5p/HMGA2/NF-κB in microglia.

### Let-7b-5p antagomir reversed rTMS effects on microglial polarization

**Fig. 6:**
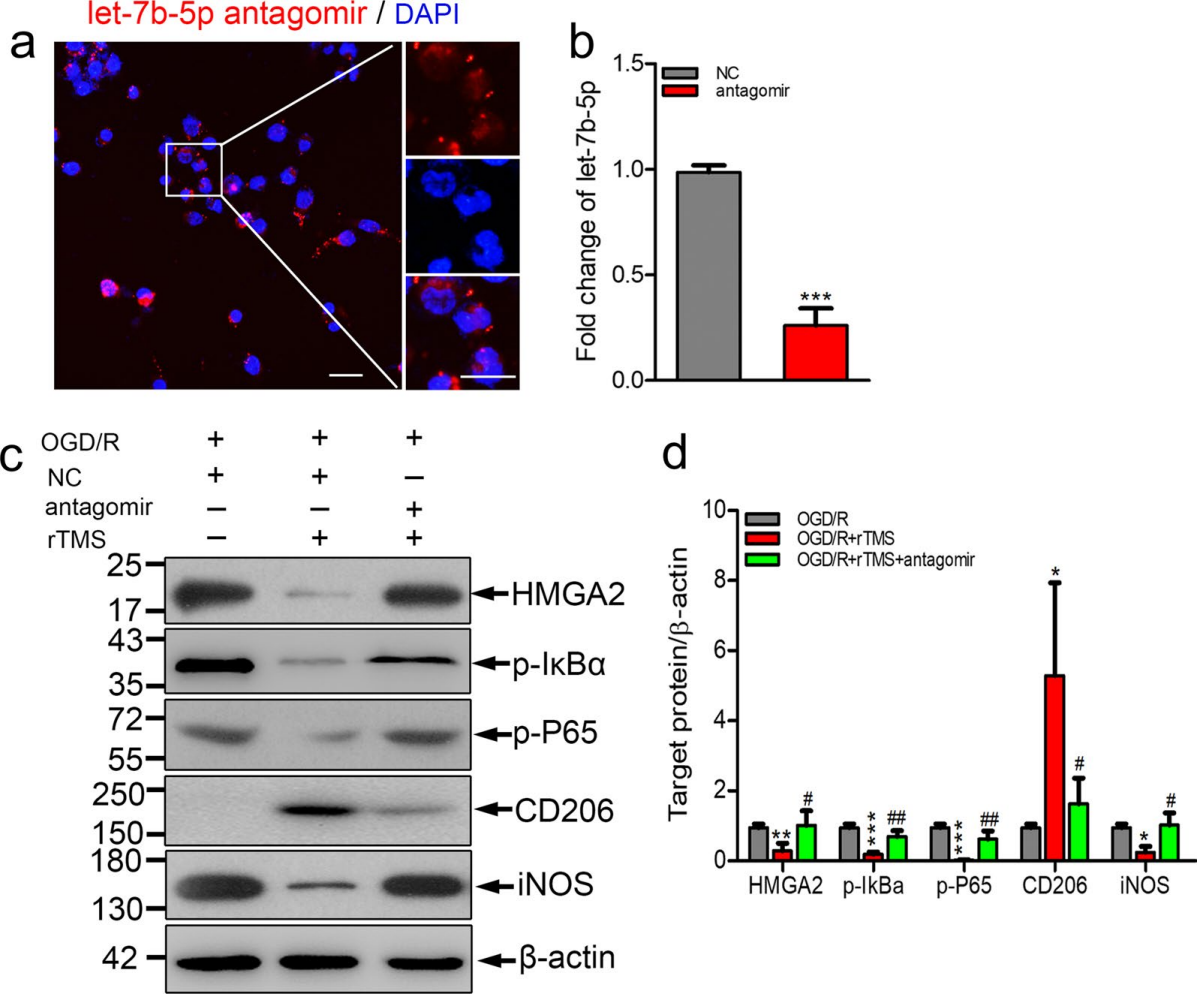
Let-7b-5p antagomir abolished rTMS effects on HMGA2/NF-κB pathway and microglia polarization. **a** Immunofluorescence staining to detect the successful transfection of Cy5-let-7b-5p antagomir (red), nucleus was in blue. **b** Real-time PCR for the detection of let-7b-5p level after NC antagomir or let-7b-5p antagomir transfection in BV2 cells (n = 3. ***P < 0.001 versus NC antagomir group). **c**, **d** Western blots and quantification of HMGA2/ NF-κB pathway associated proteins and microglia polarization markers in NC antagomir or let-7b-5p antagomir transfected OGD/R injured BV2 cells with or without rTMS treatment. n = 3. Data are expressed as mean ± SD; *P < 0.05, **P < 0.01, ***P < 0.001 versus OGD/R group; ^#^*P* < 0.05, ^##^*P* < 0.01 versus OGD/R + rTMS group. Scale bar: 5 μm

**Fig. 7:**
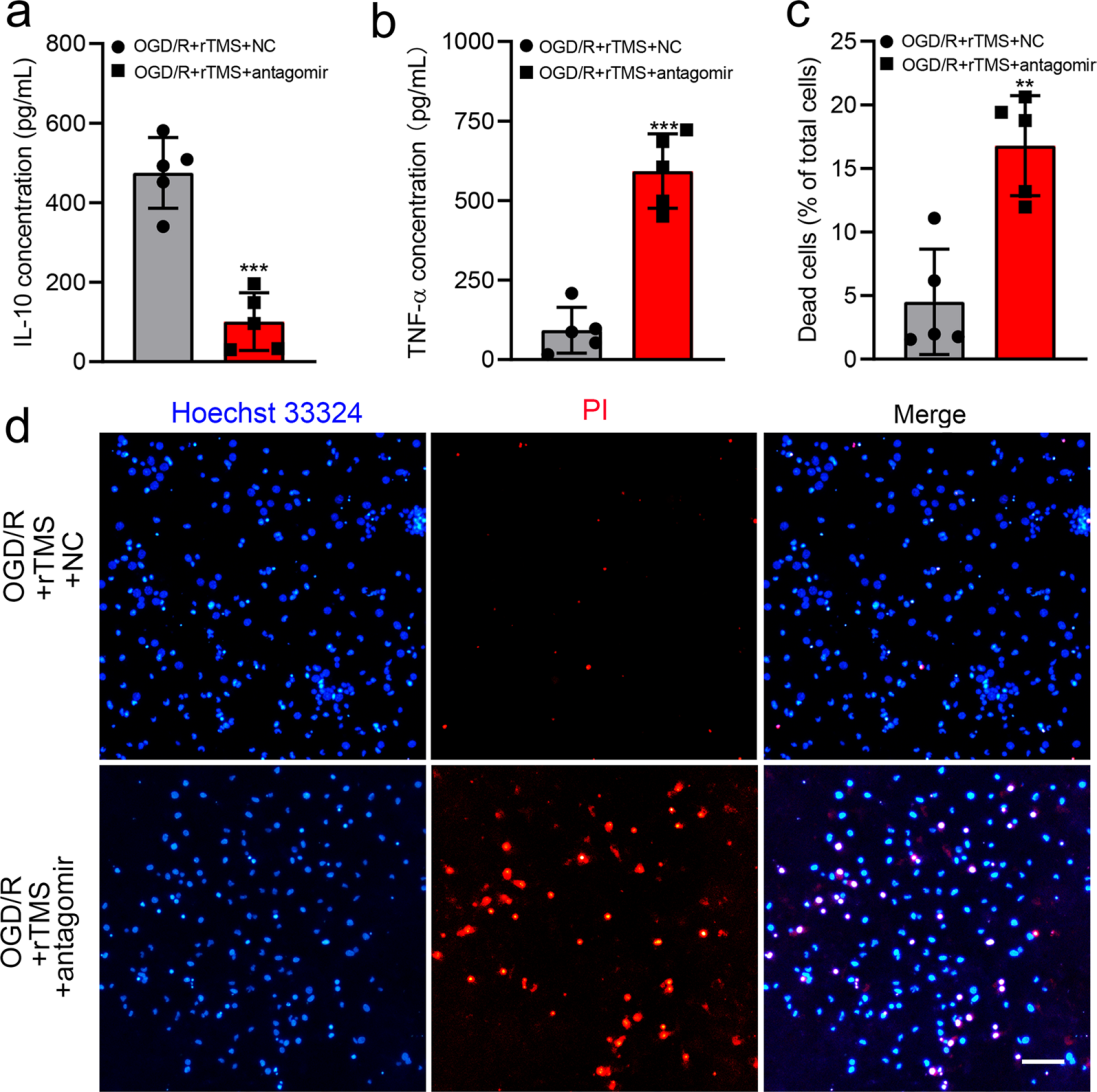
Let-7b-5p antagomir reversed neural protective roles of rTMS treated microglia. Concentration of IL-10 (**a**) and TNF-α (**b**) in different MCM. **c**, **d** Representative PI/Hoechst staining and quantification of PI positive dead neurons co-cultured with indicated MCM. n = 5. Data are expressed as mean ± SD; **P < 0.01, ***P < 0.001 versus OGD/R + rTMS + NC group. *NC* negative control, *MCM* microglia cell culture. Scale bar: 10 μm

To explore whether rTMS could regulate microglia polarization through let-7b-5p/HMGA2/NF-κB signaling pathway, let-7b-5p antagomir was used. Successfully transfected let-7b-5p antagomir mainly distributed in the cytoplasm of microglia, close to the nucleus (Fig. 6a). Transfection of let-7b-5p antagomir significantly decreased the expression of let-7b-5p in BV2 cells (Fig. 6b). rTMS significantly inhibited OGD/R induced protein levels of HMGA2, p-P65, p-IκBα and M1 phenotype marker iNOS, but increased the protein levels of microglia M2 phenotype marker CD206 in BV2 cells with negatively control antagomir transfection (Fig. 6c and d). However, when let-7b-5p expression was reduced by antagomir, rTMS effects on HMGA2, p-P65, p-IκBα expression and M2 microglial polarization were reversed. rTMS induced release of IL-10 was reduced by let-7b-5p antagomir transfection (from 474.9 to 101 pg/mL, *P* < 0.001), while TNF-α was provoked (from 92.47 to 592.6 pg/mL, *P* < 0.001) (Fig. 7a and b). As expected, MCM collected from let-7b-5p antagomir transfected cells indued more neuronal death than OGD/R + rTMS + NC antagomir group (16.79 ± 3.93% versus 4.51 ± 4.15%, *P* = 0.001) (Fig. 7c and d). Overall, rTMS could inhibit OGD/R induced M1 polarization of microglia by regulating let-7b-5p/HMGA2/NF-κB signaling pathway.

### rTMS stimulated MCM attenuated neurological deficits following ischemic stroke but counteracted by let-7b-5p antagomir

**Fig. 8:**
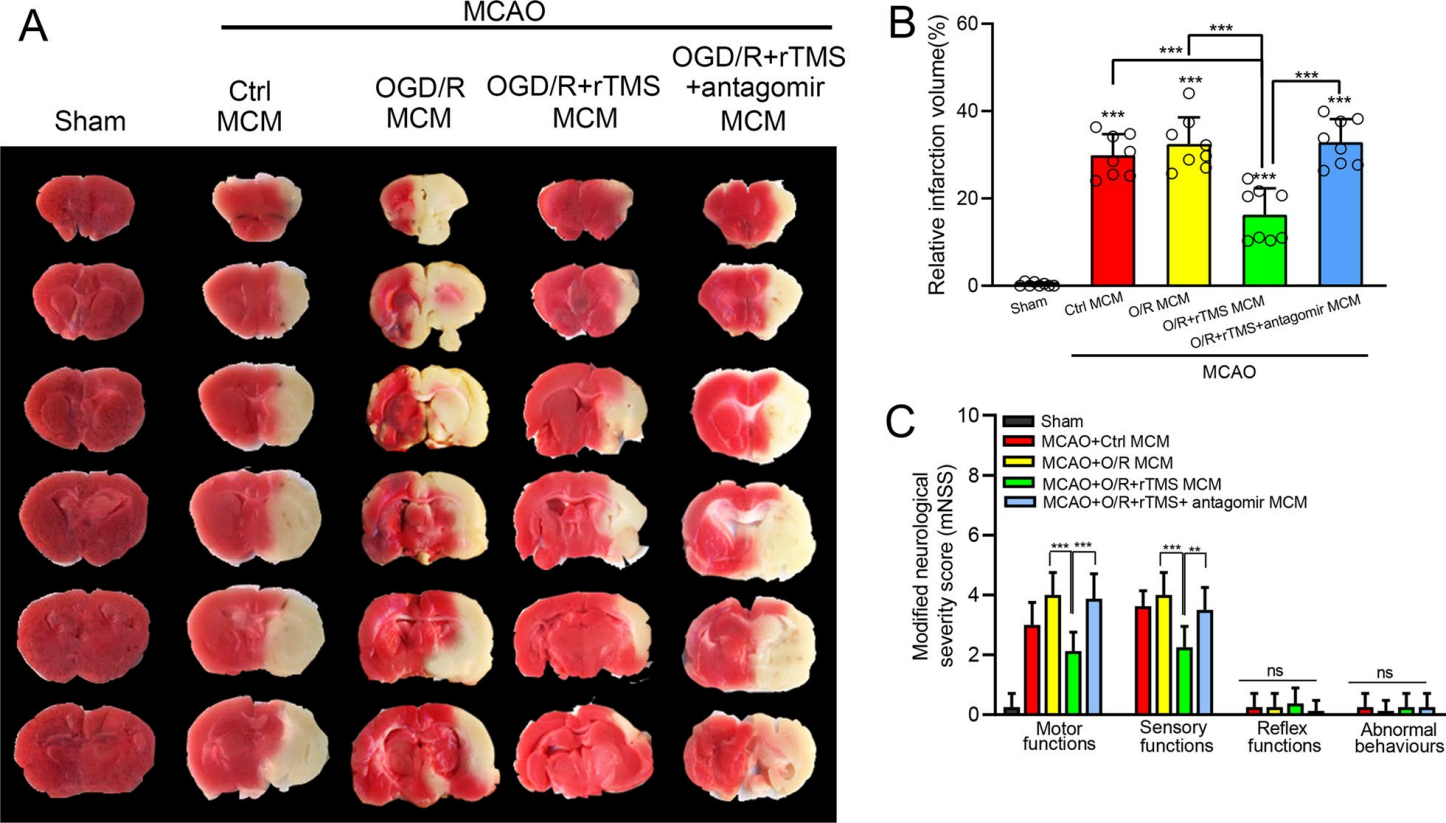
rTMS stimulated MCM reduced infarct volumes and promoted functional recovery in MCAO mice, but weaken by let-7b-5p antagomir. Immediately after reperfusion and at 3 days post reperfusion, the MCAO mice received retroorbital intravenously injection of indicated MCM. At 7 days post reperfusion, ischemic regions were detected by TTC staining. **a** Representative image of TTC staining in the indicated groups. **b** Quantitative analysis of infarct volume. **c** The neurological deficit was evaluated by modified Neurological Severity Score (mNSS). n = 8 animals per group. Data are expressed as mean ± SD; **P < 0.01, ***P < 0.001, *ns* no significance

Finally, whether rTMS-regulated MCM could still exhibit neuroprotective effect in vivo was explored. MCAO mice were randomly subjected into ctrl MCM, OGD/R MCM, OGD/R + rTMS MCM, OGD/R + rTMS + antagomir MCM group, receiving retroorbital intravenous injection of indicated MCM at the time of reperfusion, and at 3 days after reperfusion. As shown in Fig. 8a and b, The OGD/R MCM treatment lightly increased infarct volume, while the OGD/R + rTMS MCM administration significantly reduced infract volume from 32.46 ± 6.09% to 16.25 ± 6.09%, when compared to ctrl MCM-treated MCAO mice (*P* < 0.001). However, let-7b-5p antagomir transfection neutralized rTMS effects as the infract volume in OGD/R + rTMS + antagomir MCM group was increased to 32.88 ± 5.30%, around 2.0-fold higher than OGD/R + rTMS MCM group. Besides, neurological function was reflected by the mNSS tests consisting of four subitems. In MCAO mice, the best performances were observed in mice received the OGD/R + rTMS MCM. Distinguishable differences were detected in motor and sensory functions, which could reflect complex behavioral deficits. However, the benefit effects of the OGD/R + rTMS MCM disappeared in the absence of let-7b-5p, which induced by let-7b-5p antagomir (Fig. 8c).

## Discussion

This study demonstrated that 10 Hz rTMS could inhibit M1 polarization of microglia in peri-infarct area through regulating let-7b-5p/HMGA2/NF-κB signaling pathway, reducing microglia-associated neuroinflammation. The 10-Hz rTMS increased let-7b-5p to inhibit HMGA2 protein expression, and reduced NF-κB activation, resulting in reduced secretion of pro-inflammatory mediators from microglia. Down-regulation of let-7b-5p counteracted rTMS effects on microglia polarization and functional recovery after cerebral ischemic stroke.

Microglia are highly plastic cells that assume diverse phenotypes in response to the stimulations that alter their environments [3, 4]. The present study was in accordance with previous study, revealing an M2-to-M1 microglia phenotype shift in the peri-infarct region after ischemic stroke [5]. Both in mice and rats, increased CD206^+^ M2 microglia were observed in the peri-infarct region within 7 days after MCAO reperfusion, and then gradually decreased. In contrast, CD16/32^+^ or iNOS^+^ M1 microglia appeared and gradually increased on the 3rd day after MCAO reperfusion, and maintained high expression on the 14th day. Such microglia phenotype transformation has been reported in models of spinal cord injury [21], indicating that the phenotypic changes of microglia might be a common pathological mechanism of a variety of central nervous system injuries. Maintaining microglia in M2 phenotype while inhibiting M1 microglia polarization holds great potential to alleviated brain injuries after cerebral ischemic stroke.

rTMS is a promising approach for treating ischemic stroke [6]. Clinical studies confirmed rTMS effects to alleviate post-stroke dysphagia [22], aphasia [23], motor dysfunctions [24, 25], and chronic pain [26]. Despite the profound effects of rTMS to treat ischemic stroke, the cellular and molecular substrates that underlie the effects of rTMS remain poorly understood [8]. Regulation of neuronal activity is the best-known mechanism of rTMS, and different frequencies of rTMS have opposite effects. High-frequency rTMS (≥ 5 Hz) could improve neuron excitatory, while low-frequency rTMS (≤ 1 Hz) inhibits neuron excitatory, thus high-frequency rTMS always applied to lesioned cortical areas and low-frequency rTMS applied to the contralateral brain [27]. Over the past 2 decades, increasing researches have indicated that non-neuronal cells could be the likely cellular effectors of rTMS, such as astrocytes and microglia [9]. Our previous study proved that continuous application of 10-min daily 10 Hz rTMS to MCAO rats for seven days inhibited neurotoxic transformation of astrocytes [12]. However, the rTMS effects on microglia has been largely unexplored. rTMS was reported to have no effect on microglia number in the motor cortex or hippocampus in normal healthy rats [28]. On the contrary, rTMS activated microglia and increased Iba-1 expression following ischemic injury or the induction of demyelination [10, 29]. Besides, there was study indicated that high intensity and high frequency rTMS applied to the injure spinal cord attenuated microglia activation [11]. The present study found that the 10 Hz rTMS used to inhibit neurotoxic astrocytes could also prevent M1 polarization of microglia. This finding provides strong evidence that microglia is one of the cellular effectors of rTMS.

In our previous study, possible molecular mechanism by which rTMS regulated astrocytic phenotype in ischemic stroke rats was not explored [12]. As increasing evidence revealed important role of microRNAs (miRNAs) to regulate microglia activation or polarization [13, 30], miRNAs changes were detected in the current study. Both in vivo and in vitro results demonstrated that rTMS increased let-7b-5p expression in microglia. Let-7 was the first miRNA family discovered in human and highly conserved across species [31]. Let-7b is a member of the let-7 family that has been shown to exert multiple functions, such as inhibiting neural stem cell proliferation, accelerating neural differentiation and protecting mesenchymal stem cells from apoptosis and autophagy following implantation into infarcted myocardium [32, 33]. Let-7b was also reported to modulate inflammation in microglial cells, although the conclusions were inconsistent. Mukherjee et al. revealed that let-7a/b can interact with TLR7 and NOTCH signaling pathway and enhance TNF-α release from microglia [34]. However, Han et al. demonstrated that overexpression of let-7b inhibited hippocampal microglia activation, inflammation response and epileptic seizures by targeting Stat3 [35]. Controversial roles of let-7b in microglia may be explained by the different cellular context and signaling through different PPRs mediates differential expression of let-7b [34]. In the current study, let-7b-5p induced by rTMS treatment inhibited OGD/R injury triggered inflammation in microglia and reduced TNF-α release from microglia. The inhibition of let-7b-5p by antagomir impaired the rTMS effects on microglia and promoted microglia secretion of pro-inflammatory mediators to damage neurons. These results demonstrated that the function of the rTMS to regulate microglia phenotype depended on the up-regulation of the let-7b-5p.

To further elaborate the downstream pathway mediating the rTMS and let-7b-5p in microglia, TargetScan prediction and dual-luciferase reporter gene assays were applied to figure out the let-7b-5p target protein. It was confirmed that HMGA2 was one of the target genes of let-7b-5p, which is consistent with previous reports [36]. In OGD/R injured microglia, HMGA2 was significantly increased while rTMS reduced HMGA2 expression to the baseline level. However, inhibition of let-7b-5p re-increased HMGA2 in microglia. Thus, rTMS could regulate HMGA2 through modulating let-7b-5p. HMGA2 has been reported to play an important role in tumorigenesis, cell proliferation, cell transformation, and inflammation [37]. Elevation of HMGA2 notably promoted the release of proinflammatory cytokines (TNF-α, IL-6, and IL-1b), while downregulation of HMGA2 remarkably attenuated the release of proinflammatory cytokines, suggesting that HMGA2 positively regulates inflammation [38]. Besides, HMGA2 could interact with PI3K/Akt pathway to further activate NF-κB-mediated inflammatory responses [39, 40]. NF-κB activation was associated with M1 polarization of microglia [20]. In the current study, rTMS reduced p-IκBα, p-P65 expression in OGD/R microglia and reduced microglia secretion of TNF-α, while let-7b-5p antagomir transfection counteracted above rTMS effects. rTMS treatment alleviated toxicity of microglia culture media collected from OGD/R microglia to neurons. But increased neural death was detected when let-7b-5p expression was inhibited. Thus, rTMS inhibited activity of HMGA2/NF-κB pathway by increasing let-7b-5p to keep OGD/R injured microglia in M2 phenotype.

Collectively, the current study for the first to link the therapeutic effect of rTMS on ischemic stroke with microglial polarization and reveal the possible molecular mechanism was associated with let-7b-5p/HMGA2/NF-κB pathway. However, the regulation of HMGA2/NF-κB by rTMS through let-7b-5p was only assessed with in vitro model, further experiments with animals or even clinical trials are needed in the future study.

## Conclusions

The present study demonstrated that 10 Hz rTMS could alleviate neuroinflammation through inhibiting M1 polarization of microglia after cerebral ischemia–reperfusion injury. The underlying mechanism for high-frequency rTMS to modulate microglial phenotypes is associated with the upregulation of let-7b-5p to inhibit HMGA2 and NF-κB signaling pathway. This study was the first to explore rTMS effects and the possible underlying mechanisms on microglia in cerebral ischemic injury models which may extend our understanding on mechanisms of rTMS in treating ischemic stroke.

## Supplementary Information

**Additional file 1: Figure S1.** Changes of microglial polarization phenotypes in the peri-infarct region after MCAO reperfusion. Representative immunofluorescent staining of CD206 (**a**) and iNOS (**b**) in Iba-1^+^ cells on the brain sections obtained from ischemic rats at 1, 3,5,7, and 14 days after middle cerebral artery occlusion (MCAO)reperfusion or from sham-operated animals. Scale bar: 10μm.

**Additional file 2: Figure S2. **Unedited blots for Figure 2c, Figure 5c and Figure 6c.

**Additional file 3: Table S1.** Sequences of the primers used for detecting miRNAs by qRT-PCR.

**Additional file 4: Table S2.** The differential expressed miRNAs in the peri-infarct region at 7 days post-modeling.

**Additional file 5: Table S3.** Mature sequencesof the top six miRNAsincreased by rTMS treatment.

## Data Availability

The datasets supporting the conclusions of this article are included within the article and its additional files. The miRNA sequencing data discussed in this publication have been deposited in NCBI's Gene Expression Omnibus and are accessible through GEO Series Accession Number GSE192910. (https://www.ncbi.nlm.nih.gov/geo/query/acc.cgi?acc=GSE192910).

## Abbreviations

rTMS: Repetitive transcranial magnetic stimulation
MCAO: Middle cerebral artery occlusion
I/R: Ischemia–reperfusion
OGD/R: Oxygen and glucose deprivation/reoxygenation
MCM: Microglia culture medium
mNSS: Modified neurologic severity score
FISH: Fluorescence in situ hybridization
ELISA: Enzyme-linked immunosorbent assay
qRT-PCR: Quantitative real-time reverse transcription PCR
