# Supplementary material for: High-frequency repetitive transcranial magnetic stimulation (rTMS) protects against ischemic stroke by inhibiting M1 microglia polarization through let-7b-5p/HMGA2/NF-κB signaling pathway
Source: BMC Neurosci. 2022 Aug 4;23:49. doi: 10.1186/s12868-022-00735-7 (PMC9351069; doi:10.1186/s12868-022-00735-7)
Supplement: Supplementary file 1 — Additional file 1: Figure S1. Changes of microglial polarization phenotypes in the peri-infarct region after MCAO reperfusion. Representative immunofluorescent staining of CD206 (a) and iNOS (b) in Iba-1+ cells on the brain sections obtained from ischemic rats at 1, 3,5,7, and 14 days after middle cerebral artery occlusion (MCAO)reperfusion or from sham-operated animals. Scale bar: 10μm. [file 12868_2022_735_MOESM1_ESM.pdf]

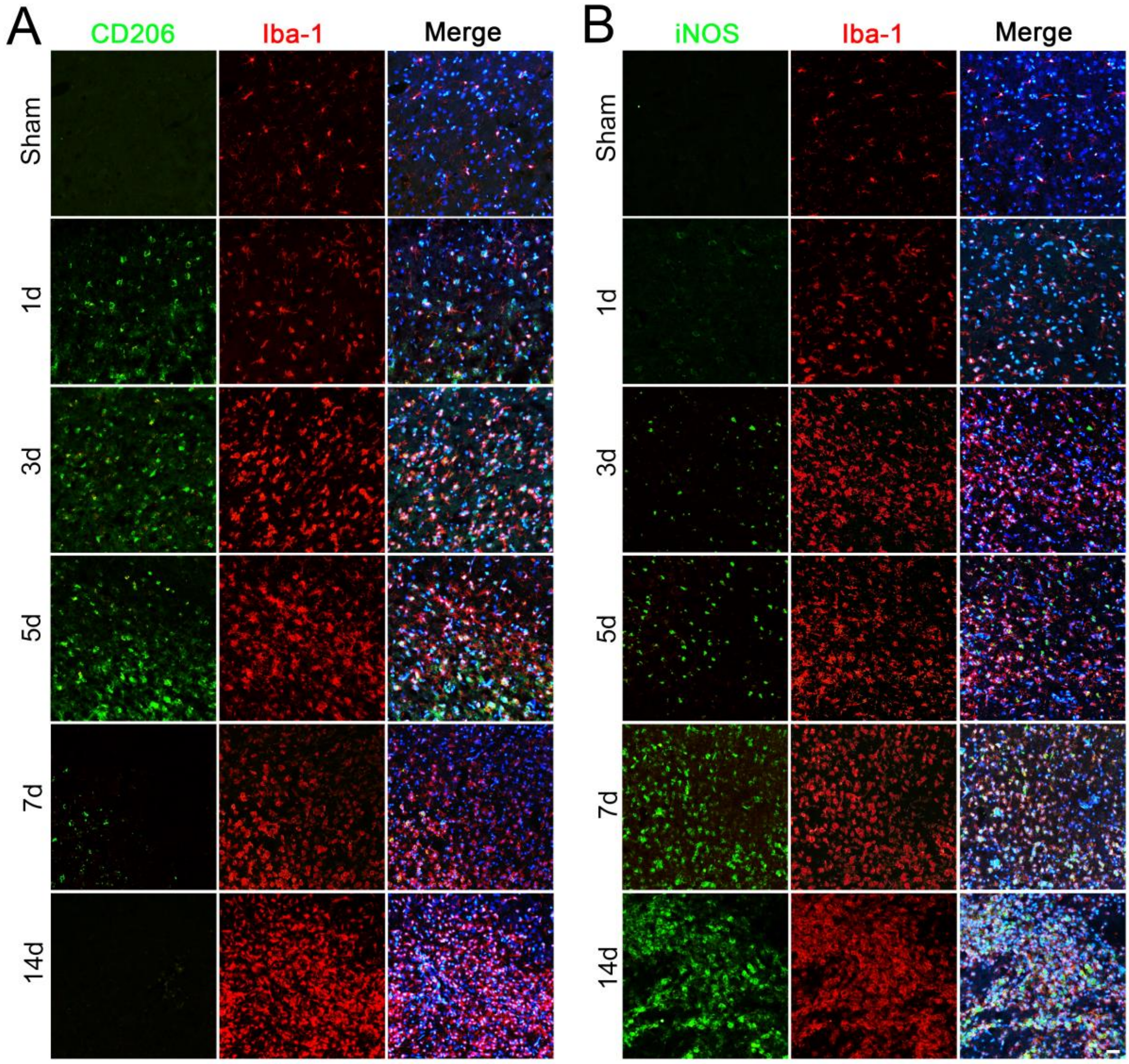

Fig. S1. Changes of microglial polarization phenotypes in the peri-infarct region after MCAO reperfusion. Representative immunofluorescent staining of CD206 (**a**) and iNOS (**b**) in Iba-1^{+} cells on the brain sections obtained from ischemic rats at 1, 3, 5, 7, and 14 days after middle cerebral artery occlusion (MCAO) reperfusion or from sham-operated animals. Scale bar: 10μm**.**
