# Supplementary material for: High-frequency repetitive transcranial magnetic stimulation (rTMS) protects against ischemic stroke by inhibiting M1 microglia polarization through let-7b-5p/HMGA2/NF-κB signaling pathway
Source: BMC Neurosci. 2022 Aug 4;23:49. doi: 10.1186/s12868-022-00735-7 (PMC9351069; doi:10.1186/s12868-022-00735-7)

Unedited blots for Figure 2C

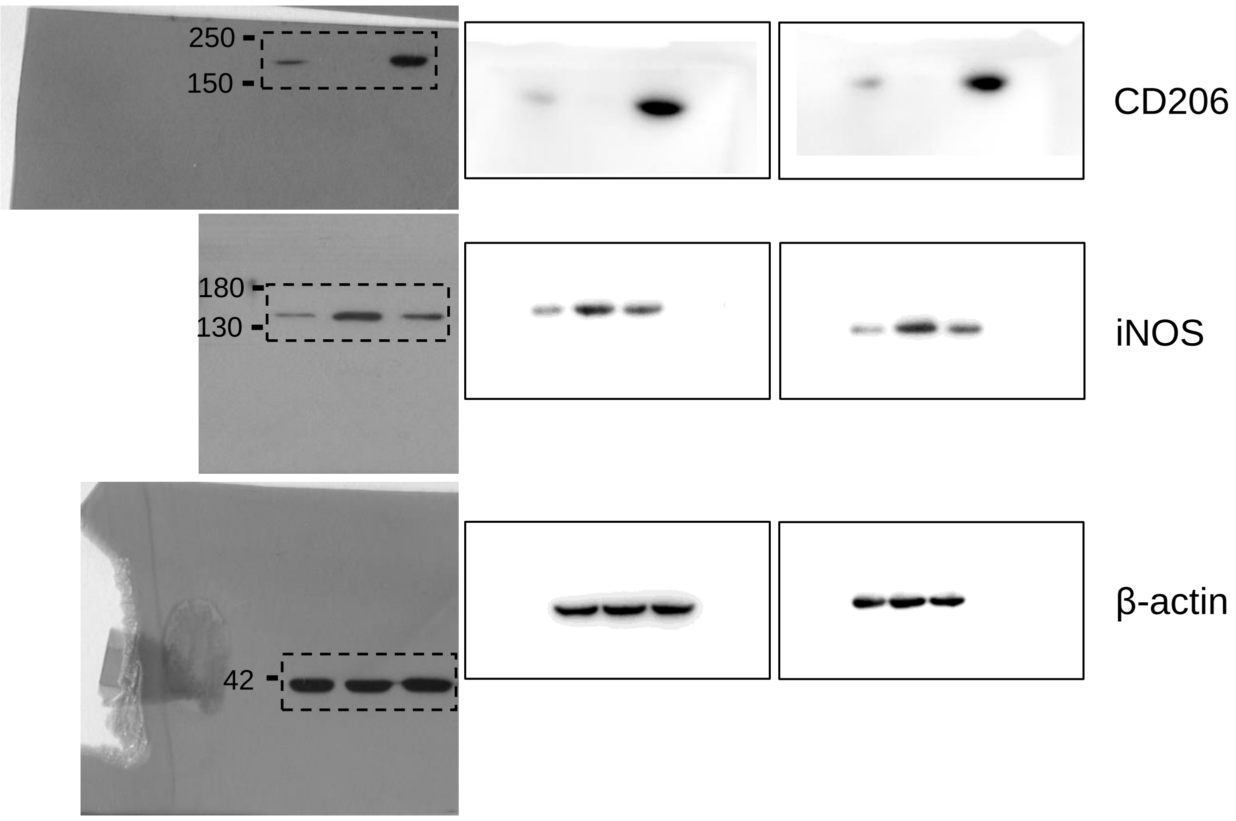

Blots with high contrast

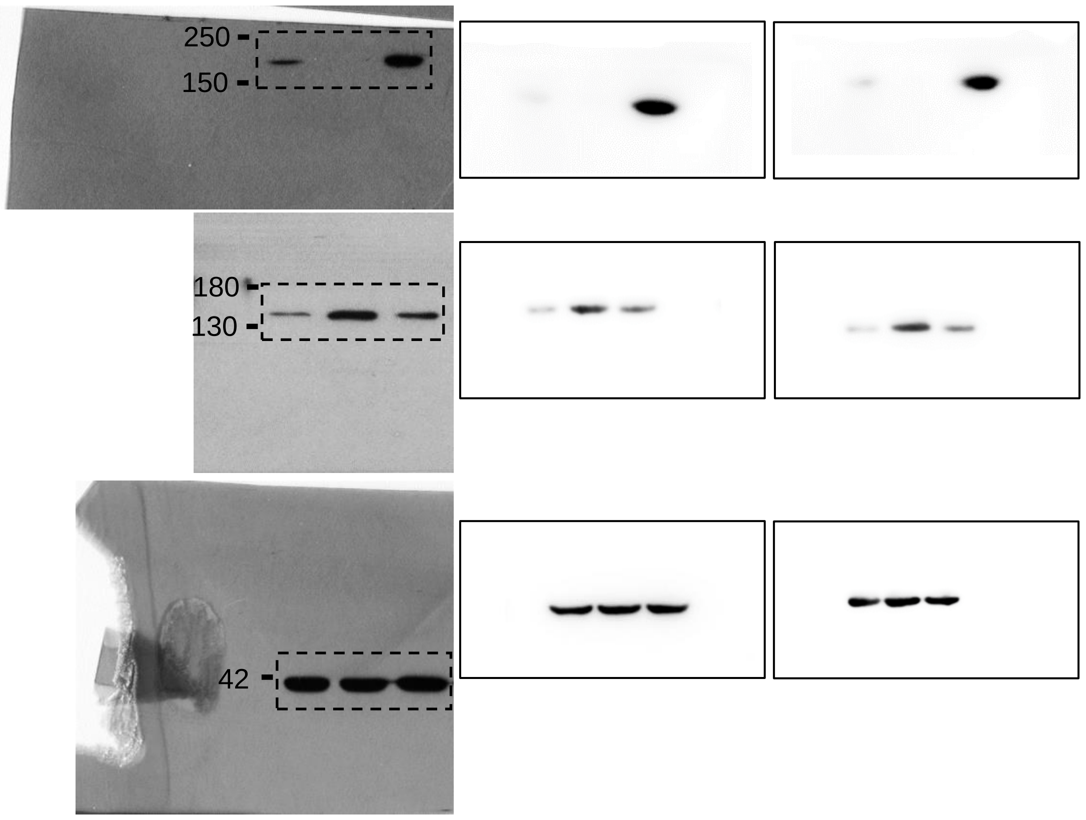

Blots with low contrast

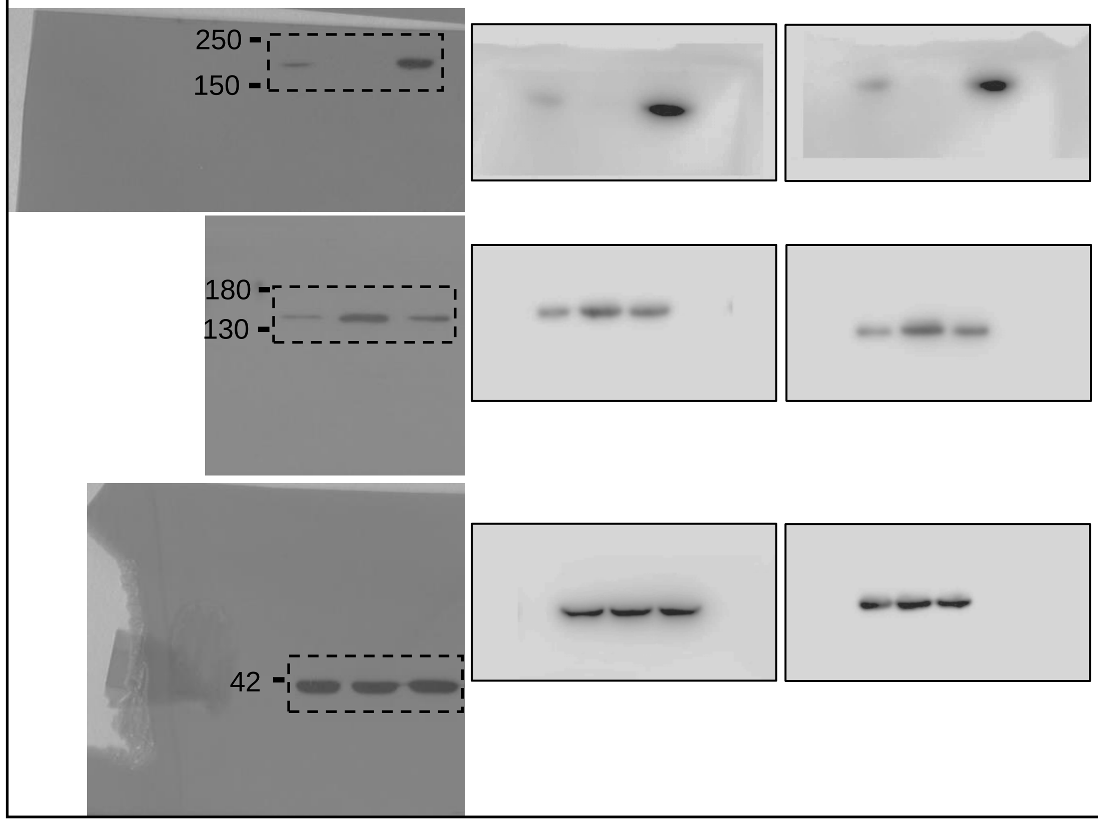

Unedited blots for Figure 5C

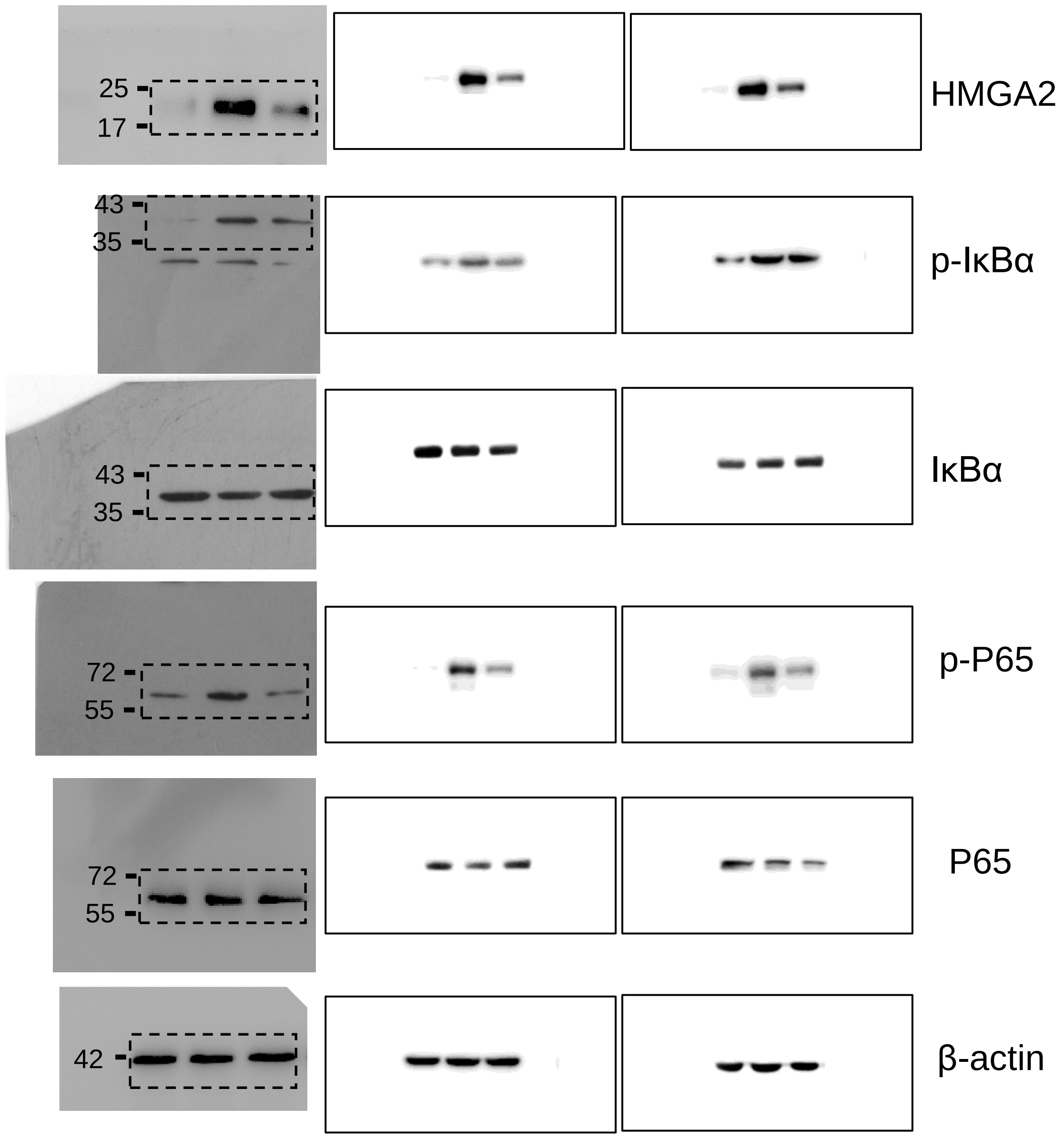

Blots with high contrast

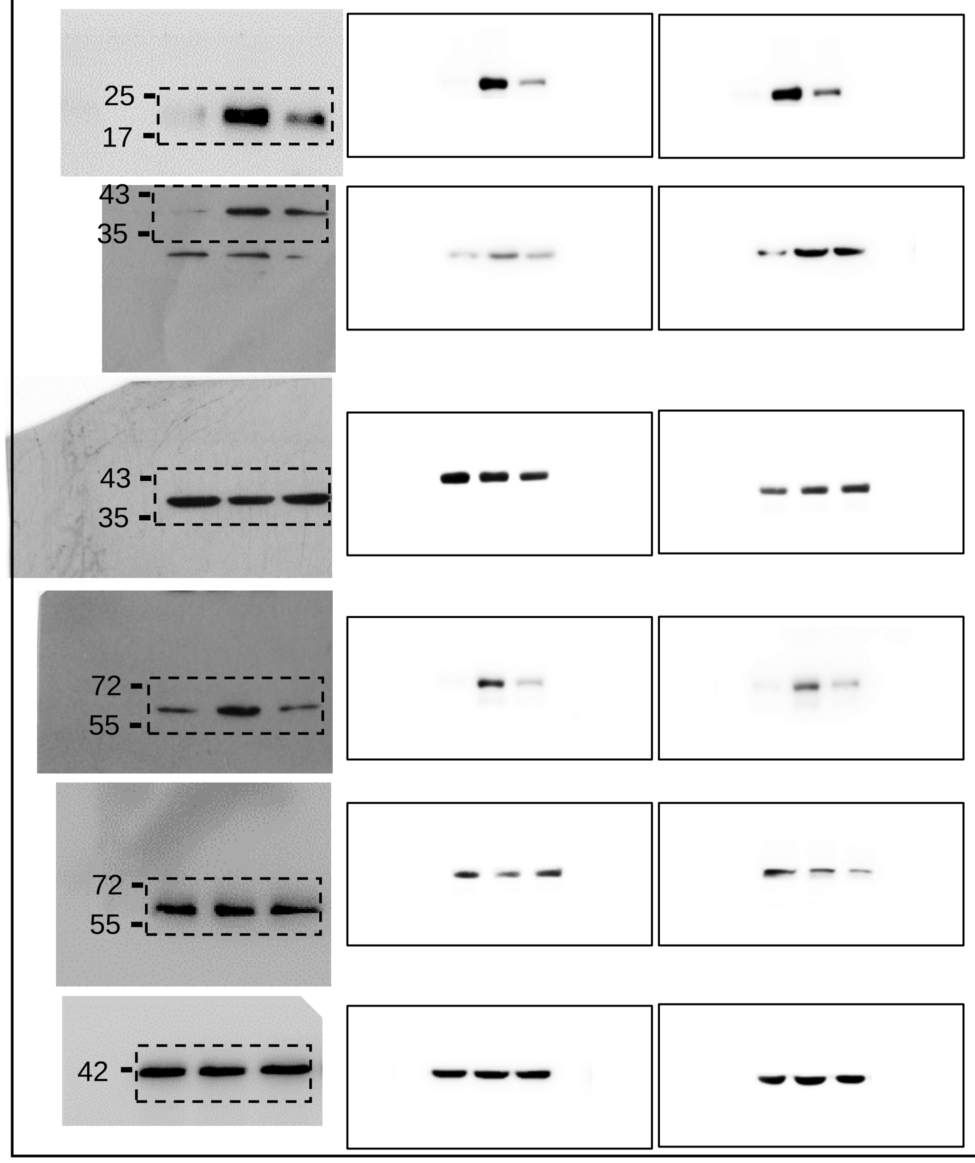

Blots with low contrast

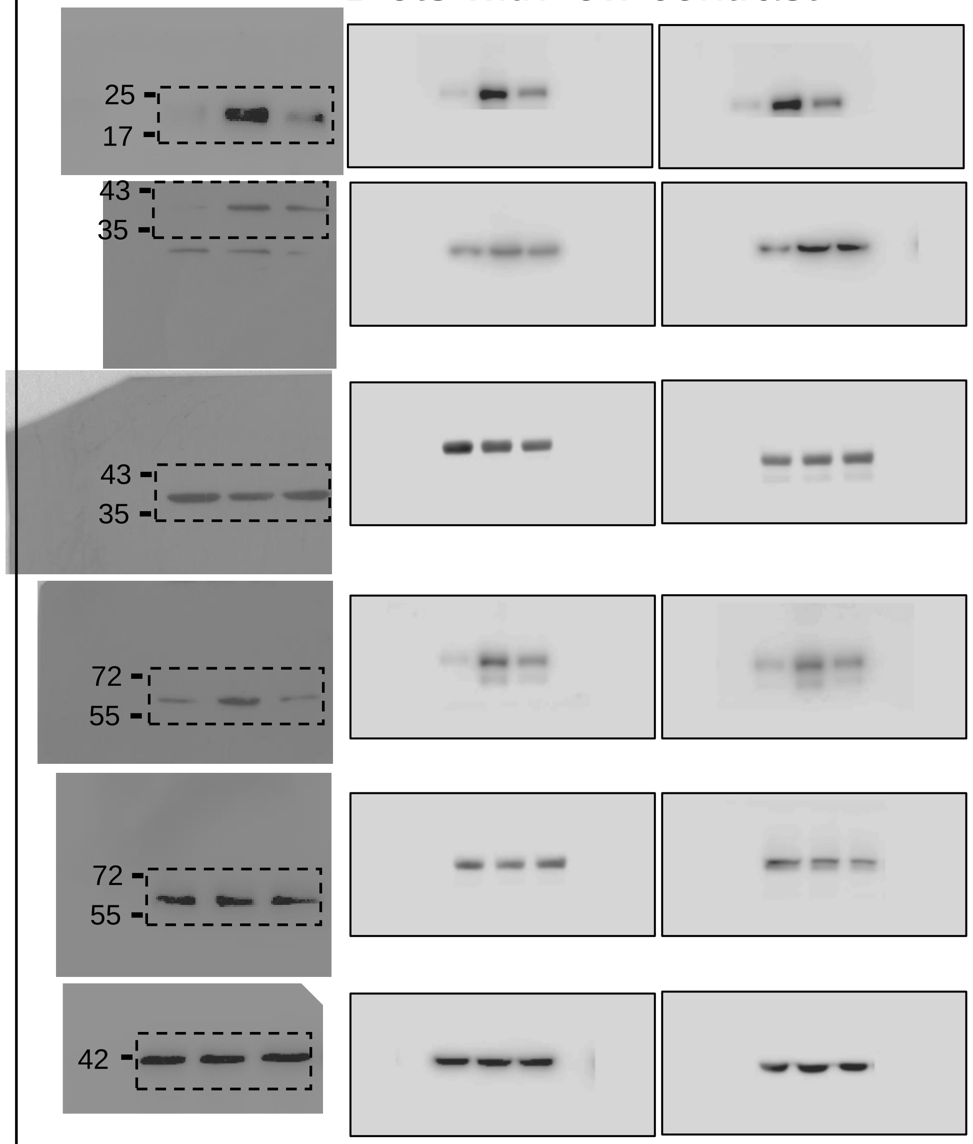

Unedited blots for Figure 6C

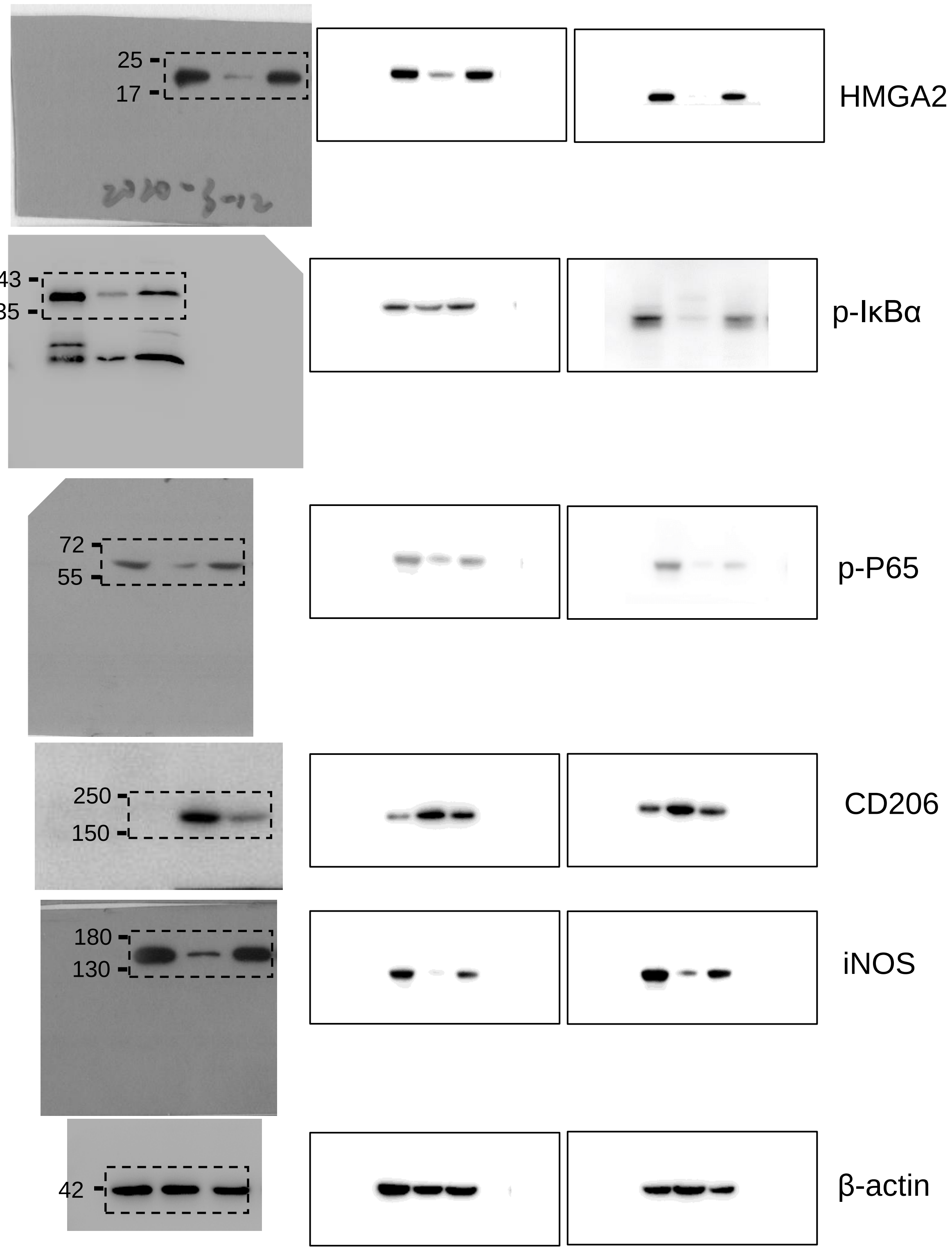

Blots with high contrast

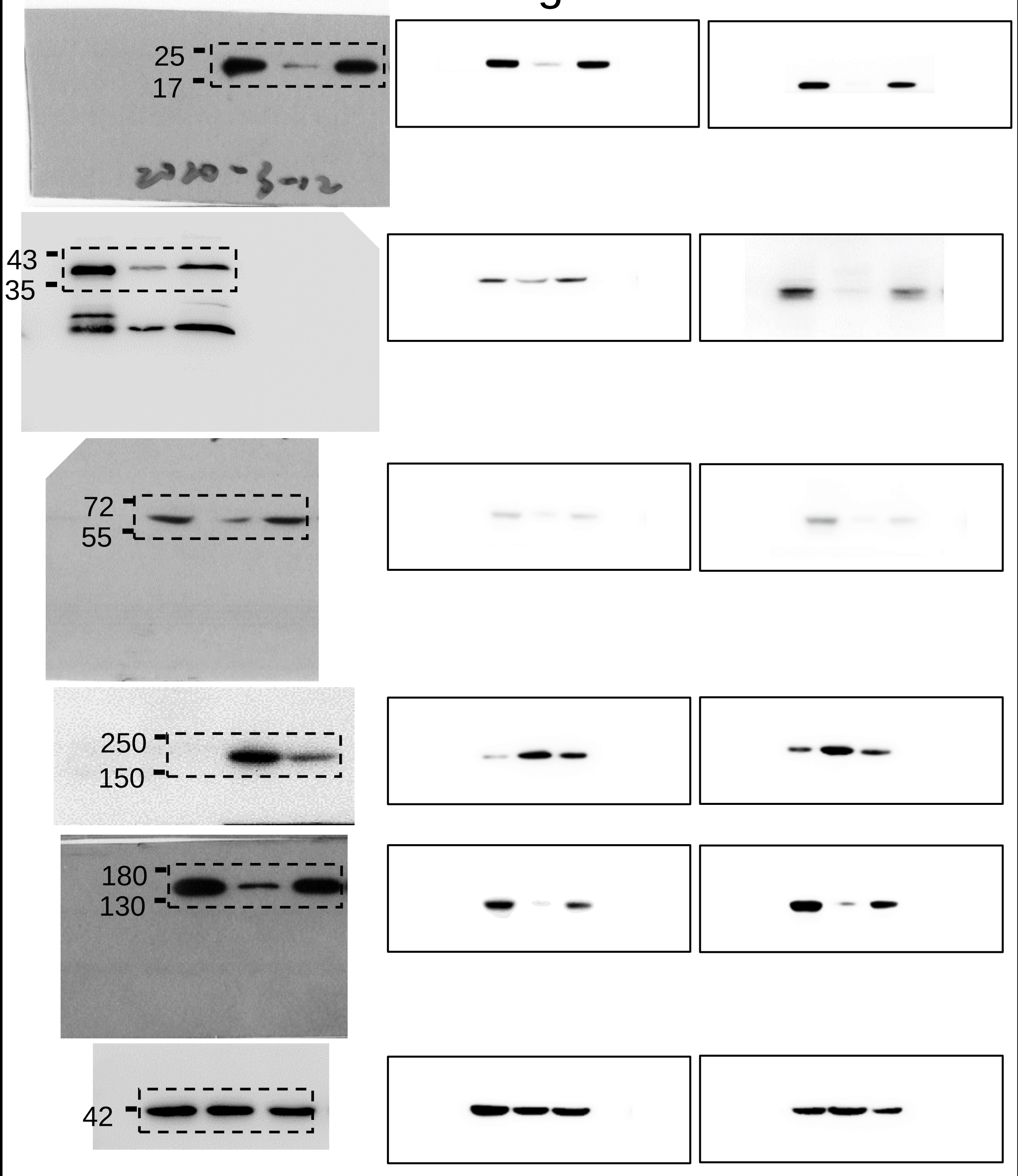

Blots with low contrast

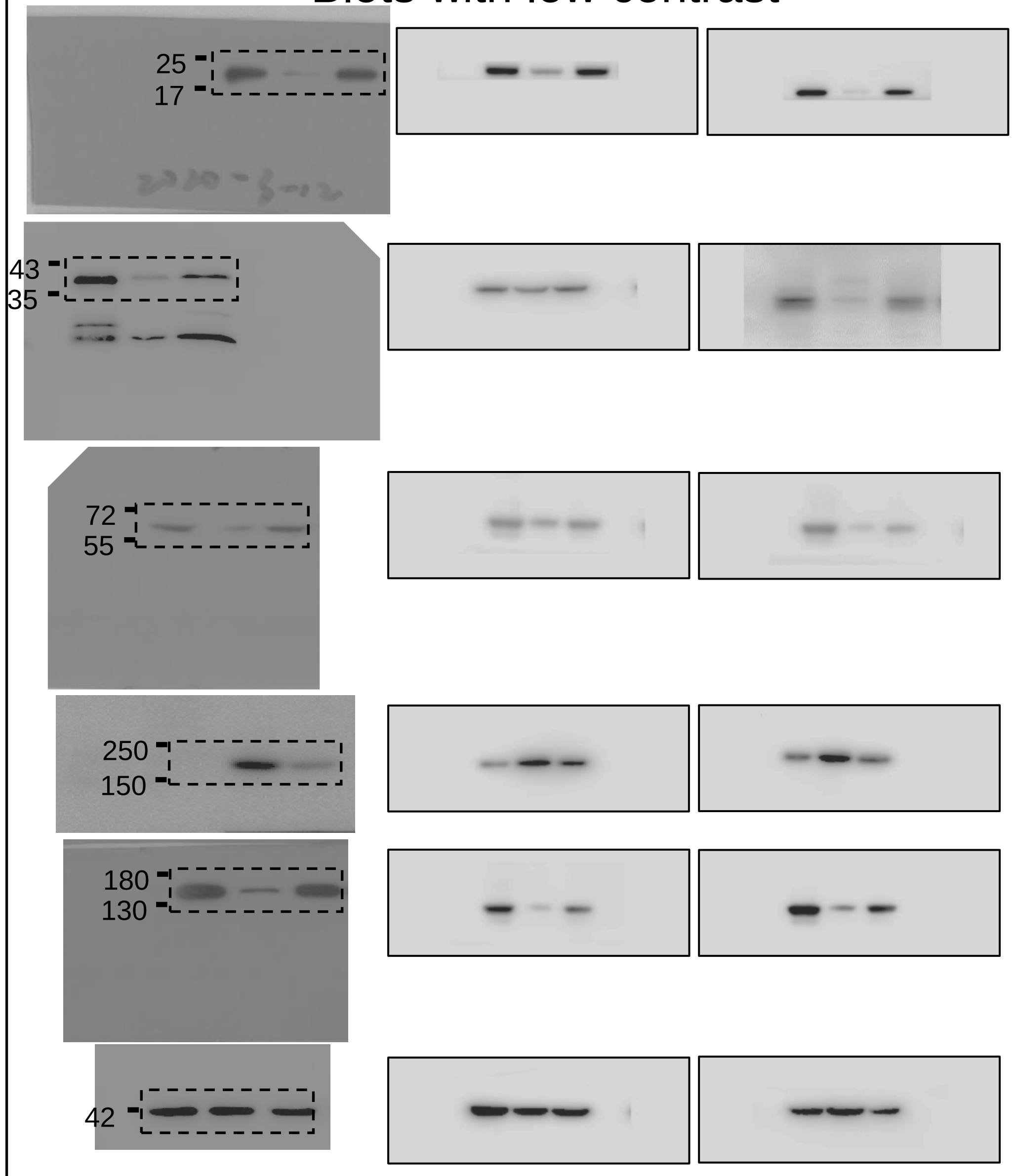

Supplement: Supplementary file 2 — Additional file 2: Figure S2. Unedited blots for Figure 2c, Figure 5c and Figure 6c. [file 12868_2022_735_MOESM2_ESM.pdf]
