# Supplementary material for: High-frequency repetitive transcranial magnetic stimulation (rTMS) protects against ischemic stroke by inhibiting M1 microglia polarization through let-7b-5p/HMGA2/NF-κB signaling pathway
Source: BMC Neurosci. 2022 Aug 4;23:49. doi: 10.1186/s12868-022-00735-7 (PMC9351069; doi:10.1186/s12868-022-00735-7)
Supplement: Supplementary file 3 — Additional file 3: Table S1. Sequences of the primers used for detecting miRNAs by qRT-PCR. [file 12868_2022_735_MOESM3_ESM.pdf]

Table S1. Sequences of the primers used for detecting miRNAs by qRT-PCR.

miRNAs	Forward primer (5'-3')	Reverse primer (5'-3')
let-7b-5p	ATCCAGTGCGTGTCGTG	TGCTTGAGGTAGTAGGTTG
let-7c-5p	GGGGTGAGGTAGTAGGTTGT	CAGTGCAGGGTCCGAGGT
miR-206-3p	CTGCCGTGGAATGTAAGGAA	TATGGTTGTTCTGCTCTCTGTCTC
miR-671-3p	CTGGCTGGACAGAGTTGTCAT	TCCGGTTCTCAGGGCTCCACC
miR-1224	CTCTCATGTGAGGACTGGGGA	AGTGCGTGTCGTGGAGTCG
U6	TGCGGGTGCTCGCTTCGGCAGC	CCAGTGCAGGGTCCGAGGT
