# Supplementary material for: High-frequency repetitive transcranial magnetic stimulation (rTMS) protects against ischemic stroke by inhibiting M1 microglia polarization through let-7b-5p/HMGA2/NF-κB signaling pathway
Source: BMC Neurosci. 2022 Aug 4;23:49. doi: 10.1186/s12868-022-00735-7 (PMC9351069; doi:10.1186/s12868-022-00735-7)
Supplement: Supplementary file 4 — Additional file 4: Table S2. The differential expressed miRNAs in the peri-infarct region at 7 days post-modeling. [file 12868_2022_735_MOESM4_ESM.pdf]

**Table S2. The differential expressed miRNAs in the peri-infarct region at 7 days post-modeling.**

| miRNA | MCAO-1 | MCAO-2 | MCAO-3 | MCAO-rTMS-1 | MCAO-rTMS-2 | MCAO-rTMS-3 | Fold_change | p-value |
|---|---|---|---|---|---|---|---|---|
| rno-let-7f-5p | 414341 | 329267 | 361266 | 876937 | 806718 | 585768 | 2.607673514 | 8.82E-07 |
| rno-miR-127-3p | 382201 | 319011 | 349485 | 683535 | 648815 | 723964 | 2.533515285 | 3.51E-06 |
| rno-let-7c-5p | 332781 | 302811 | 413350 | 1790997 | 1696664 | 1999134 | 6.798388899 | 6.84E-16 |
| rno-let-7a-5p | 263268 | 219404 | 294873 | 902115 | 830677 | 599422 | 3.815132402 | 2.08E-11 |
| rno-let-7i-5p | 203021 | 176182 | 177695 | 330741 | 299871 | 325786 | 2.208527874 | 0.000126954 |
| rno-let-7b-5p | 52087 | 50376 | 58391 | 294695 | 291108 | 320171 | 7.264233078 | 3.31E-19 |
| rno-miR-143-3p | 50704 | 30605 | 25595 | 84919 | 47477 | 51472 | 2.192035832 | 0.000268097 |
| rno-miR-99b-5p | 47750 | 43246 | 52702 | 94424 | 95181 | 136272 | 2.978145682 | 0.001130298 |
| rno-let-7e-5p | 40300 | 36089 | 45645 | 147443 | 144935 | 116819 | 4.280821981 | 3.60E-11 |
| rno-miR-9a-3p | 37785 | 19559 | 21315 | 8868 | 5470 | 6103 | 0.336724691 | 1.20E-05 |
| rno-miR-212-5p | 19666 | 15082 | 22158 | 38656 | 38440 | 60570 | 3.223700856 | 0.003524068 |
| rno-miR-323-3p | 19010 | 13132 | 20012 | 40136 | 33806 | 19412 | 2.268299764 | 0.000140273 |
| rno-miR-379-5p | 18434 | 11774 | 10885 | 27354 | 20647 | 22818 | 2.227317712 | 0.000296612 |
| rno-miR-378a-3p | 17919 | 14818 | 17667 | 32771 | 30881 | 34914 | 2.537550895 | 3.08E-05 |
| rno-miR-219a-2-3p | 11843 | 9266 | 8898 | 24019 | 24819 | 9787 | 2.431720558 | 0.003008687 |
| rno-miR-92a-3p | 10843 | 9629 | 12787 | 22811 | 21887 | 16364 | 2.336852901 | 9.47E-05 |
| rno-miR-30e-5p | 10584 | 8221 | 12526 | 2675 | 2758 | 2351 | 0.321562964 | 1.03E-07 |
| rno-miR-151-3p | 10476 | 8540 | 9906 | 28018 | 26327 | 52722 | 4.950108754 | 0.006853694 |
| rno-miR-361-3p | 9147 | 9036 | 9521 | 12471 | 15112 | 18251 | 2.155349632 | 0.005779205 |
| rno-miR-186-5p | 9108 | 7122 | 11254 | 4435 | 3625 | 1537 | 0.435635197 | 0.000157766 |
| rno-miR-138-5p | 8980 | 6922 | 8420 | 2087 | 2094 | 2052 | 0.332158291 | 1.44E-07 |
| rno-miR-382-5p | 8902 | 6888 | 11822 | 15566 | 13778 | 14913 | 2.090032294 | 0.000970088 |
| rno-miR-1843b-5p | 8647 | 5815 | 5726 | 12894 | 10068 | 14729 | 2.448418277 | 0.000661114 |
| rno-miR-92b-3p | 8075 | 7607 | 12527 | 26360 | 29031 | 27986 | 3.840076502 | 2.84E-09 |
| rno-miR-409a-3p | 7598 | 6817 | 8812 | 12541 | 12596 | 11132 | 2.00466162 | 0.001388778 |
| rno-miR-30e-3p | 7061 | 5351 | 5646 | 15573 | 12090 | 11735 | 2.789328365 | 2.65E-06 |
| rno-miR-107-3p | 6969 | 5569 | 7716 | 2899 | 2403 | 988 | 0.385960729 | 1.07E-05 |
| rno-miR-25-3p | 6019 | 5060 | 6466 | 10298 | 8617 | 8509 | 2.005665571 | 0.001177605 |
| rno-miR-1843a-5p | 5949 | 4088 | 4094 | 9250 | 7290 | 10573 | 2.514264935 | 0.000400983 |
| rno-miR-487b-3p | 5242 | 3870 | 5994 | 1814 | 1488 | 1093 | 0.371171628 | 1.82E-06 |
| rno-miR-369-5p | 4840 | 3307 | 3730 | 1341 | 976 | 1312 | 0.398133367 | 4.72E-06 |
| rno-miR-30a-3p | 4508 | 3915 | 4108 | 15712 | 12757 | 13982 | 4.340153492 | 5.07E-11 |
| rno-miR-342-3p | 4317 | 4512 | 6006 | 1840 | 2547 | 1391 | 0.493670551 | 0.000693466 |
| rno-miR-30b-5p | 3876 | 2677 | 1706 | 1082 | 649 | 193 | 0.28167657 | 0.00150061 |
| rno-miR-485-5p | 3792 | 3251 | 4119 | 13282 | 12277 | 13892 | 4.581938808 | 1.39E-11 |
| rno-miR-770-3p | 3291 | 1666 | 3654 | 4454 | 5010 | 6959 | 2.591409381 | 0.008851067 |
| rno-miR-16-5p | 3262 | 2324 | 2554 | 1053 | 744 | 499 | 0.354426474 | 8.18E-07 |
| rno-miR-17-5p | 3179 | 2363 | 2891 | 765 | 644 | 238 | 0.242211215 | 4.12E-11 |
| rno-miR-29b-3p | 3122 | 2197 | 1926 | 1149 | 600 | 435 | 0.373618895 | 2.64E-05 |
| rno-miR-384-5p | 3082 | 2177 | 2951 | 1102 | 800 | 500 | 0.368487556 | 1.96E-06 |
| rno-miR-320-3p | 3072 | 4139 | 4613 | 5812 | 8015 | 6389 | 2.172246729 | 0.00021309 |
| rno-miR-93-5p | 2954 | 2664 | 3607 | 1291 | 1218 | 484 | 0.402908662 | 1.82E-05 |
| rno-miR-325-5p | 2798 | 1863 | 1897 | 3914 | 2995 | 3405 | 2.035618766 | 0.000577773 |
| rno-miR-485-3p | 2614 | 2022 | 2701 | 8829 | 9963 | 12033 | 5.538293794 | 7.44E-08 |
| rno-miR-378b | 2611 | 1871 | 2106 | 4704 | 4386 | 4209 | 2.60990528 | 4.31E-06 |
| rno-miR-410-3p | 2585 | 1590 | 2832 | 1198 | 788 | 450 | 0.438728617 | 9.04E-05 |
| rno-miR-134-5p | 2409 | 1746 | 2770 | 3833 | 3681 | 3739 | 2.121401046 | 0.000334889 |
| rno-miR-194-5p | 2392 | 1656 | 1897 | 475 | 341 | 233 | 0.222470233 | 4.24E-12 |
| rno-miR-344b-3p | 2390 | 1398 | 1472 | 348 | 298 | 292 | 0.231777849 | 8.66E-12 |
| rno-miR-99b-3p | 2264 | 1878 | 2608 | 3435 | 3452 | 4415 | 2.199065893 | 0.001389833 |
| rno-miR-486 | 2192 | 2922 | 2065 | 6683 | 6579 | 6441 | 3.43209068 | 3.83E-09 |
| rno-miR-708-3p | 2031 | 1091 | 748 | 2612 | 1851 | 2792 | 2.468599904 | 0.002357653 |
| rno-miR-24-2-5p | 1977 | 1242 | 1125 | 524 | 353 | 644 | 0.462459586 | 0.001236907 |
| rno-miR-423-5p | 1887 | 1886 | 2572 | 5872 | 6321 | 4489 | 3.343042483 | 7.89E-09 |
| rno-miR-125b-1-3p | 1711 | 1213 | 1740 | 4580 | 4456 | 4896 | 3.913064387 | 1.69E-10 |
| rno-miR-199a-3p | 1642 | 989 | 1805 | 3186 | 3027 | 1179 | 2.110713599 | 0.013342183 |
| rno-miR-139-3p | 1511 | 1131 | 1829 | 5132 | 5306 | 5071 | 4.525823382 | 2.10E-12 |
| rno-miR-30c-2-3p | 1477 | 1284 | 1274 | 2815 | 2303 | 2180 | 2.29879971 | 3.62E-05 |
| rno-miR-126a-5p | 1460 | 769 | 958 | 451 | 383 | 274 | 0.448259247 | 0.000195262 |
| rno-miR-140-5p | 1432 | 1051 | 1185 | 352 | 280 | 262 | 0.312194893 | 9.21E-08 |
| rno-miR-135a-5p | 1374 | 858 | 1755 | 207 | 213 | 153 | 0.186960874 | 5.45E-11 |
| rno-miR-6331 | 1335 | 1091 | 2050 | 4770 | 4681 | 3690 | 3.788624117 | 2.28E-10 |
| rno-miR-494-3p | 1220 | 1075 | 1664 | 594 | 589 | 149 | 0.415132962 | 0.000492468 |
| rno-miR-708-5p | 1217 | 1127 | 1783 | 562 | 574 | 250 | 0.420748281 | 0.000102217 |
| rno-miR-106b-3p | 1172 | 996 | 1202 | 2451 | 2135 | 2012 | 2.507121097 | 6.80E-06 |
| rno-miR-322-3p | 1036 | 532 | 630 | 1632 | 1408 | 566 | 2.073419803 | 0.018432414 |
| rno-miR-339-5p | 1019 | 855 | 1299 | 365 | 364 | 95 | 0.321131529 | 6.10E-07 |
| rno-miR-20a-5p | 1002 | 753 | 932 | 217 | 228 | 63 | 0.234580766 | 6.46E-10 |
| rno-miR-135b-5p | 1000 | 583 | 579 | 113 | 106 | 245 | 0.292746908 | 7.31E-06 |
| rno-miR-195-5p | 975 | 687 | 656 | 254 | 150 | 216 | 0.344865181 | 2.38E-06 |
| rno-miR-493-5p | 956 | 744 | 987 | 2670 | 2298 | 1795 | 3.210572941 | 1.39E-08 |
| rno-miR-152-3p | 858 | 584 | 969 | 2321 | 1904 | 1317 | 2.937274765 | 1.65E-07 |
| rno-miR-155-5p | 825 | 634 | 1285 | 311 | 385 | 274 | 0.460037744 | 0.001667056 |
| rno-miR-181c-5p | 789 | 565 | 931 | 282 | 194 | 228 | 0.399477338 | 3.80E-05 |

| | | | | | | | | |
|---|---|---|---|---|---|---|---|---|
| rno-miR-1224 | 786 | 649 | 731 | 2577 | 2478 | 3468 | 5.161558548 | 2.24E-06 |
| rno-miR-671 | 775 | 740 | 772 | 2281 | 2579 | 4128 | 5.18674368 | 0.001077455 |
| rno-miR-151-5p | 762 | 468 | 844 | 1798 | 1420 | 945 | 2.569454709 | 5.29E-06 |
| rno-miR-1249 | 758 | 523 | 844 | 1061 | 1164 | 1206 | 2.126183714 | 0.000496436 |
| rno-miR-380-3p | 757 | 452 | 518 | 254 | 188 | 202 | 0.483695429 | 0.001361729 |
| rno-miR-497-5p | 734 | 612 | 632 | 198 | 133 | 92 | 0.266327375 | 3.86E-08 |
| rno-miR-29c-3p | 702 | 448 | 668 | 238 | 198 | 124 | 0.392492952 | 6.45E-05 |
| rno-miR-412-5p | 692 | 446 | 761 | 1248 | 1101 | 1071 | 2.350334373 | 6.59E-05 |
| rno-miR-499-5p | 630 | 330 | 368 | 134 | 146 | 141 | 0.418430484 | 0.000190167 |
| rno-miR-1b | 625 | 478 | 589 | 2226 | 1580 | 1399 | 3.909112534 | 7.41E-11 |
| rno-miR-384-3p | 624 | 316 | 366 | 116 | 73 | 61 | 0.245335933 | 1.12E-07 |
| rno-miR-206-3p | 619 | 1000 | 491 | 3054 | 3963 | 2054 | 5.197051672 | 2.19E-08 |
| rno-miR-376b-3p | 574 | 322 | 328 | 121 | 80 | 69 | 0.282046782 | 4.99E-07 |
| rno-miR-1843a-3p | 574 | 493 | 612 | 1428 | 1259 | 659 | 2.490919316 | 2.41E-05 |
| rno-miR-376a-5p | 518 | 325 | 473 | 96 | 68 | 52 | 0.209426856 | 9.85E-10 |
| rno-miR-873-5p | 503 | 322 | 341 | 926 | 555 | 656 | 2.354551032 | 9.25E-05 |
| rno-miR-133a-3p | 490 | 549 | 697 | 320 | 227 | 140 | 0.488543991 | 0.003007517 |
| rno-miR-369-3p | 477 | 269 | 437 | 117 | 118 | 37 | 0.290290212 | 1.28E-06 |
| rno-miR-873-3p | 451 | 319 | 556 | 978 | 874 | 666 | 2.444732516 | 3.63E-05 |
| rno-miR-31a-5p | 429 | 282 | 534 | 112 | 119 | 27 | 0.259300979 | 1.52E-07 |
| rno-miR-136-5p | 425 | 273 | 361 | 67 | 74 | 150 | 0.374483905 | 3.08E-05 |
| rno-miR-128-2-5p | 386 | 341 | 329 | 1016 | 975 | 1299 | 4.051928638 | 2.43E-06 |
| rno-miR-137-3p | 361 | 226 | 427 | 41 | 86 | 51 | 0.232094904 | 1.46E-08 |
| rno-miR-301a-5p | 327 | 206 | 243 | 119 | 77 | 100 | 0.495391989 | 0.005247817 |
| rno-miR-322-5p | 306 | 230 | 252 | 98 | 70 | 36 | 0.321471424 | 3.01E-05 |
| rno-miR-872-3p | 286 | 231 | 316 | 116 | 80 | 31 | 0.335320078 | 5.65E-05 |
| rno-miR-323-5p | 279 | 209 | 296 | 1013 | 906 | 1011 | 4.870163886 | 2.09E-12 |
| rno-miR-664-2-5p | 273 | 246 | 362 | 2070 | 2332 | 2416 | 10.08731355 | 1.97E-20 |
| rno-miR-7a-1-3p | 257 | 167 | 178 | 86 | 57 | 28 | 0.353141583 | 0.000243852 |
| rno-miR-342-5p | 254 | 263 | 316 | 483 | 567 | 646 | 2.644878703 | 0.000131773 |
| rno-miR-125a-3p | 254 | 216 | 271 | 846 | 915 | 947 | 4.74879122 | 5.79E-12 |
| rno-miR-296-5p | 237 | 163 | 333 | 93 | 88 | 33 | 0.368393852 | 0.0002658 |
| rno-miR-130a-3p | 205 | 170 | 254 | 93 | 57 | 56 | 0.415606897 | 0.001240509 |
| rno-miR-184 | 200 | 169 | 149 | 519 | 554 | 631 | 4.264295654 | 3.12E-09 |
| rno-miR-672-5p | 198 | 207 | 382 | 1592 | 1672 | 1381 | 7.596064187 | 7.41E-20 |
| rno-let-7i-3p | 197 | 124 | 191 | 55 | 38 | 39 | 0.333291153 | 0.000113463 |
| rno-miR-30c-1-3p | 194 | 175 | 181 | 421 | 358 | 287 | 2.446991207 | 0.000136998 |
| rno-miR-425-3p | 181 | 141 | 227 | 91 | 67 | 54 | 0.491671037 | 0.009707489 |
| rno-miR-128-1-5p | 178 | 126 | 162 | 325 | 303 | 260 | 2.457483333 | 0.000254179 |
| rno-miR-362-5p | 177 | 150 | 183 | 13 | 27 | 0 | 0.095881063 | 2.66E-12 |
| rno-miR-181a-2-3p | 161 | 159 | 153 | 602 | 601 | 264 | 3.829818627 | 9.24E-06 |
| rno-miR-130b-5p | 158 | 120 | 127 | 268 | 288 | 139 | 2.16030766 | 0.001613397 |
| rno-miR-106b-5p | 151 | 107 | 119 | 25 | 13 | 10 | 0.158683363 | 6.96E-08 |
| rno-miR-223-3p | 142 | 147 | 222 | 61 | 51 | 14 | 0.301088789 | 6.30E-05 |
| rno-miR-200c-3p | 134 | 95 | 194 | 62 | 71 | 29 | 0.487704697 | 0.013532752 |
| rno-miR-92b-5p | 133 | 156 | 276 | 418 | 505 | 313 | 2.784205643 | 1.28E-05 |
| rno-miR-551b-3p | 125 | 119 | 126 | 17 | 13 | 28 | 0.20689074 | 8.99E-07 |
| rno-miR-370-5p | 118 | 74 | 97 | 224 | 197 | 94 | 2.250168447 | 0.001641516 |
| rno-miR-363-3p | 111 | 114 | 98 | 51 | 43 | 23 | 0.445425603 | 0.012345137 |
| rno-miR-582-5p | 98 | 76 | 69 | 31 | 15 | 10 | 0.282577572 | 0.000653089 |
| rno-miR-1-3p | 97 | 97 | 93 | 638 | 449 | 261 | 5.768659496 | 1.83E-08 |
| rno-miR-350 | 94 | 76 | 110 | 48 | 44 | 0 | 0.394633702 | 0.008332531 |
| rno-miR-1843b-3p | 94 | 66 | 78 | 257 | 223 | 149 | 3.356836314 | 3.65E-06 |
| rno-miR-19b-3p | 86 | 56 | 70 | 10 | 0 | 16 | 0.166838894 | 6.96E-06 |
| rno-miR-15a-5p | 86 | 73 | 73 | 22 | 11 | 11 | 0.236324385 | 0.000149349 |
| rno-miR-335 | 85 | 58 | 58 | 11 | 13 | 12 | 0.233499688 | 0.000211305 |
| rno-miR-150-3p | 70 | 66 | 64 | 221 | 182 | 229 | 4.058971944 | 1.78E-07 |
| rno-miR-190a-5p | 70 | 52 | 94 | 22 | 16 | 0 | 0.21143164 | 0.000139409 |
| rno-miR-142-3p | 69 | 66 | 58 | 13 | 18 | 29 | 0.41195228 | 0.012110006 |
| rno-miR-296-3p | 68 | 59 | 110 | 162 | 171 | 91 | 2.273713296 | 0.002292843 |
| rno-miR-665 | 67 | 35 | 107 | 22 | 15 | 17 | 0.340952089 | 0.007928335 |
| rno-miR-539-3p | 65 | 36 | 94 | 17 | 15 | 20 | 0.357734522 | 0.004846986 |
| rno-miR-666-5p | 61 | 38 | 69 | 127 | 114 | 142 | 3.021304904 | 0.000168512 |
| rno-miR-93-3p | 56 | 58 | 71 | 36 | 16 | 0 | 0.326589445 | 0.008185923 |
| rno-miR-377-3p | 50 | 24 | 51 | 0 | 0 | 0 | 0 | 2.77E-09 |
| rno-miR-23b-5p | 47 | 41 | 68 | 355 | 309 | 300 | 7.971515313 | 8.86E-15 |
| rno-miR-1188-5p | 43 | 25 | 122 | 125 | 131 | 91 | 2.411686539 | 0.002822884 |
| rno-miR-99a-3p | 35 | 37 | 83 | 11 | 19 | 11 | 0.343264932 | 0.016533202 |
| rno-miR-6318 | 35 | 24 | 38 | 11 | 10 | 0 | 0.261974334 | 0.017710968 |
| rno-miR-377-5p | 35 | 33 | 57 | 15 | 0 | 0 | 0.135825336 | 0.000355549 |
| rno-miR-541-3p | 34 | 16 | 27 | 10 | 0 | 0 | 0.150568428 | 0.008340456 |
| rno-miR-153-5p | 34 | 19 | 33 | 0 | 0 | 0 | 0 | 1.59E-06 |
| rno-miR-218a-2-3p | 32 | 24 | 30 | 0 | 0 | 0 | 0 | 1.34E-06 |
| rno-miR-17-1-3p | 32 | 30 | 33 | 0 | 0 | 0 | 0 | 2.44E-07 |
| rno-miR-3542 | 30 | 16 | 30 | 12 | 0 | 0 | 0.182437781 | 0.015702567 |
| rno-miR-10b-5p | 27 | 50 | 81 | 385 | 314 | 435 | 9.202936956 | 5.42E-12 |
| rno-miR-466b-3p | 26 | 0 | 14 | 0 | 0 | 0 | 0 | 0.015146748 |
| rno-miR-300-5p | 26 | 23 | 26 | 0 | 0 | 0 | 0 | 8.77E-06 |

| | | | | | | | | |
|---|---|---|---|---|---|---|---|---|
| rno-miR-295-3p | 26 | 16 | 14 | 0 | 0 | 0 | 0 | 0.000306356 |
| rno-miR-351-3p | 24 | 12 | 40 | 46 | 56 | 46 | 2.601450519 | 0.013281142 |
| rno-miR-222-5p | 23 | 13 | 21 | 39 | 37 | 39 | 2.662501203 | 0.020523766 |
| rno-miR-376c-3p | 23 | 22 | 15 | 0 | 0 | 0 | 0 | 0.000119971 |
| rno-miR-3065-5p | 23 | 16 | 31 | 0 | 0 | 0 | 0 | 2.57E-05 |
| rno-miR-154-3p | 21 | 16 | 16 | 0 | 0 | 0 | 0 | 0.00050071 |
| rno-miR-6315 | 21 | 20 | 16 | 0 | 0 | 0 | 0 | 0.000215084 |
| rno-miR-449a-5p | 21 | 19 | 27 | 0 | 0 | 0 | 0 | 3.85E-05 |
| rno-miR-224-5p | 18 | 18 | 25 | 64 | 55 | 55 | 3.650703107 | 0.000415887 |
| rno-miR-193b-5p | 17 | 30 | 27 | 74 | 94 | 65 | 3.901254193 | 3.42E-05 |
| rno-miR-92a-1-5p | 17 | 10 | 13 | 0 | 0 | 0 | 0 | 0.006025565 |
| rno-miR-500-3p | 14 | 15 | 11 | 0 | 0 | 0 | 0 | 0.004957209 |
| rno-miR-337-3p | 13 | 12 | 23 | 0 | 0 | 0 | 0 | 0.001317901 |
| rno-miR-483-3p | 13 | 0 | 22 | 58 | 49 | 36 | 5.604775491 | 7.65E-05 |
| rno-miR-3064-5p | 13 | 13 | 25 | 36 | 45 | 34 | 2.917460381 | 0.010958795 |
| rno-miR-18a-5p | 13 | 19 | 12 | 0 | 0 | 0 | 0 | 0.002092516 |
| rno-miR-483-5p | 10 | 11 | 13 | 31 | 42 | 31 | 3.904057311 | 0.002766621 |
| rno-miR-3594-5p | 10 | 0 | 11 | 59 | 46 | 35 | 9.184434634 | 2.23E-06 |
| rno-miR-410-5p | 0 | 14 | 15 | 56 | 36 | 42 | 5.519317722 | 4.37E-05 |
| rno-miR-6321 | 0 | 0 | 142 | 184 | 224 | 308 | 7.081321091 | 3.89E-04 |
