# Supplementary material for: High-frequency repetitive transcranial magnetic stimulation (rTMS) protects against ischemic stroke by inhibiting M1 microglia polarization through let-7b-5p/HMGA2/NF-κB signaling pathway
Source: BMC Neurosci. 2022 Aug 4;23:49. doi: 10.1186/s12868-022-00735-7 (PMC9351069; doi:10.1186/s12868-022-00735-7)
Supplement: Supplementary file 5 — Additional file 5: Table S3. Mature sequencesof the top six miRNAsincreased by rTMS treatment. [file 12868_2022_735_MOESM5_ESM.pdf]

Supplementary Table 3 Mature sequences of the top six miRNAs increased by rTMS treatment**.**

**miRNAs**	**Species**	
	Rat (rno）	Mouse (mmu)
let-7b-5p	UGAGGUAGUAGGUUGUGUGGUU	UGAGGUAGUAGGUUGUGUGGUU
let-7c-5p	UGAGGUAGUAGGUUGUAUGGUU	UGAGGUAGUAGGUUGUAUGGUU
miR-485-3p	CAUACACGGCUCUCCUCUCUUC	AGUCAUACACGGCUCUCCUCUC
miR-206-3p	UGGAAUGUAAGGAAGUGUGUGG	UGGAAUGUAAGGAAGUGUGUGG
miR-671-3p	UCCGGUUCUCAGGGCUCCACC	UCCGGUUCUCAGGGCUCCACC
miR-1224	GUGAGGACUGGGGAGGUGGAG	GUGAGGACUGGGGAGGUGGAG
